# Longitudinal study of the scalp microbiome suggests coconut oil to enrich healthy scalp commensals

**DOI:** 10.1038/s41598-021-86454-1

**Published:** 2021-03-31

**Authors:** Rituja Saxena, Parul Mittal, Cecile Clavaud, Darshan B. Dhakan, Nita Roy, Lionel Breton, Namita Misra, Vineet K. Sharma

**Affiliations:** 1 Metagenomics and Systems Biology Laboratory, Academic Building 3, Department of Biological Sciences, Indian Institute of Science Education and Research Bhopal, Bhauri, Bhopal, 462066 India; 2 L’Oréal Research & Innovation, 1 Av. Eugene Schueller, 92601, Aulnay-sous-bois, France; 3 L’Oréal India Pvt. Ltd., Bengaluru, India

**Keywords:** Clinical microbiology, Microbiology

## Abstract

Dandruff is a recurrent chronic scalp disorder, affecting majority of the population worldwide. Recently a metagenomic study of the Indian scalp microbiome described an imperative role of bacterial commensals in providing essential vitamins and amino acids to the scalp. Coconut oil and its formulations are commonly applied on the scalp in several parts of the world to maintain scalp health. Thus, in this study we examined the effect of topical application of coconut oil on the scalp microbiome (bacterial and fungal) at the taxonomic and functional levels and their correlation with scalp physiological parameters. A 16-weeks-long time-course study was performed including 12-weeks of treatment and 4-weeks of relapse phase on a cohort of 140 (70 healthy and 70 dandruff) Indian women, resulting in ~ 900 metagenomic samples. After the treatment phase, an increase in the abundance of *Cutibacterium acnes* and *Malassezia globosa* in dandruff scalp was observed, which were negatively correlated to dandruff parameters. At the functional level, an enrichment of healthy scalp-related bacterial pathways, such as biotin metabolism and decrease in the fungal pathogenesis pathways was observed. The study provides novel insights on the effect of coconut oil in maintaining a healthy scalp and in modulating the scalp microbiome.

## Introduction

The human skin including the scalp surface, serves as the body’s first line of defence as well as a host to a myriad of microorganisms, which includes both bacteria and fungi^1^. The application of high-throughput next-generation sequencing and robust computational analysis has led to an in-depth understanding of the scalp microbiome in the recent years^2–4^, providing novel clues on the pathophysiology of scalp-related disorders such as dandruff and seborrheic dermatitis in different countries^5–9^. Dandruff is one of the most common scalp condition affecting majority of the population worldwide^10^. It is a recurrent, chronic, sub-inflammatory disorder, which is characterized by scaly patches and sometimes itching^10,11^. Various environmental and intrinsic factors are reported to be linked to the development of dandruff, such as the sebum composition, host susceptibility, scalp microbiome, and a combined interaction between all of these.

Global studies have revealed that the scalp microbiome is characterized by a rather low bacterial diversity, as compared to the other body sites^12,13^, and is dominated by *Cutibacterium acnes* (formerly *Propionibacterium acnes*), *Staphylococcus epidermidis* and *Malassezia* spp*.*^4–7^. *Staphylococcus epidermidis* and *Cutibacterium acnes* are found to be the key bacterial players, where dandruff is commonly marked with an increased abundance of *S. epidermidis* on the scalp^5–7^. Among the fungal microbiota, different species of *Malassezia*, specifically *M. restricta* and *M. globosa* have shown varying proportions in populations of different countries^5,14–16^. Specific strains of *M. restricta* have been identified in the dandruff patients at the genotypic level^17^. The ability of *Malassezia* sp. to metabolize and oxidize sebum-derived lipids (triglycerides, squalene, fatty acids, etc.) is an additional source of potential inflammatory compounds^18^. *M. restricta* is also known to induce cytotoxicity to skin cells in vitro, suggesting an active role in the acceleration of dandruff^19^. A strong association of uncharacterised *Malassezia* species with dandruff is also observed in a few recent studies^4,7^. Further, scalp bacteria have been reported to have a stronger association with scaling severity than fungi suggesting that bacteria could have an implication in the clinical symptoms^14,15^. However, whether the scalp microbiome variation is a cause or a consequence of the unhealthy condition of the scalp remains unclear. *S. epidermidis* and *C. acnes* are also a part of the commensal microbiota and reported to have beneficial activities on the skin through immune response modulation and protection against pathogens^20–22^. In our recent metagenomic study carried out on the scalp microflora of the Indian population, we have observed enrichment of bacterial pathways related to the synthesis and metabolism of amino acids, biotin and B-vitamins in healthy scalp compared to dandruff, revealing a new potential role of bacterial commensals in maintaining the scalp nutrient homeostasis^4^.

Current anti-dandruff therapies involve topical antifungal agents such as azoles, the clinical efficacy of which is accompanied by a reduction in the proportion of *Malassezia* spp. on the scalp^23–26^. However, it is usually observed that at the cessation of the treatment, the relapse restores the initial symptoms^24,27^. Scalp-related products such as oils, shampoo and other cosmetics are also used worldwide to maintain scalp health and hygiene^28,29^. Among which, coconut oil is the most widely used product in African and Asian countries, including India, to ameliorate scalp health and hair growth^29–31^. Not much is known about the mode of action of coconut oil. Firstly, the antifungal activity of lauric acid, the major fatty acid contained in the oil, is suspected to prevent the proliferation of pathogens^29,31–34^. Secondly, coconut oil is known to have a biophysical action on the skin barrier function, since it helps to decrease the TEWL (trans-epidermal water loss) on long-term application^35,36^. However, no study has yet systematically examined the effect of topical application of coconut oil on the scalp microbiome.

Due to the recently established role of the microbiome on skin and scalp health, a few studies have investigated the effect of emollients ^37,38^ and topical medication^39,40^ on the skin microbiome. Here, we carried out a 16-weeks-long time-course study to understand the impact of coconut oil application on the scalp microbiome (bacterial and fungal) of 140 individuals with healthy and dandruff scalp. A treatment phase was carried out for 12-weeks followed by 4-weeks of the relapse phase in which no application of oil was performed. The scalp clinical parameters were also recorded throughout the study and correlated with the taxonomic and functional profile of the scalp microbiome. The present study aims to provide insights on the potential effect of coconut oil on the scalp fungal and bacterial microbiome.

## Results

**Figure 1:**
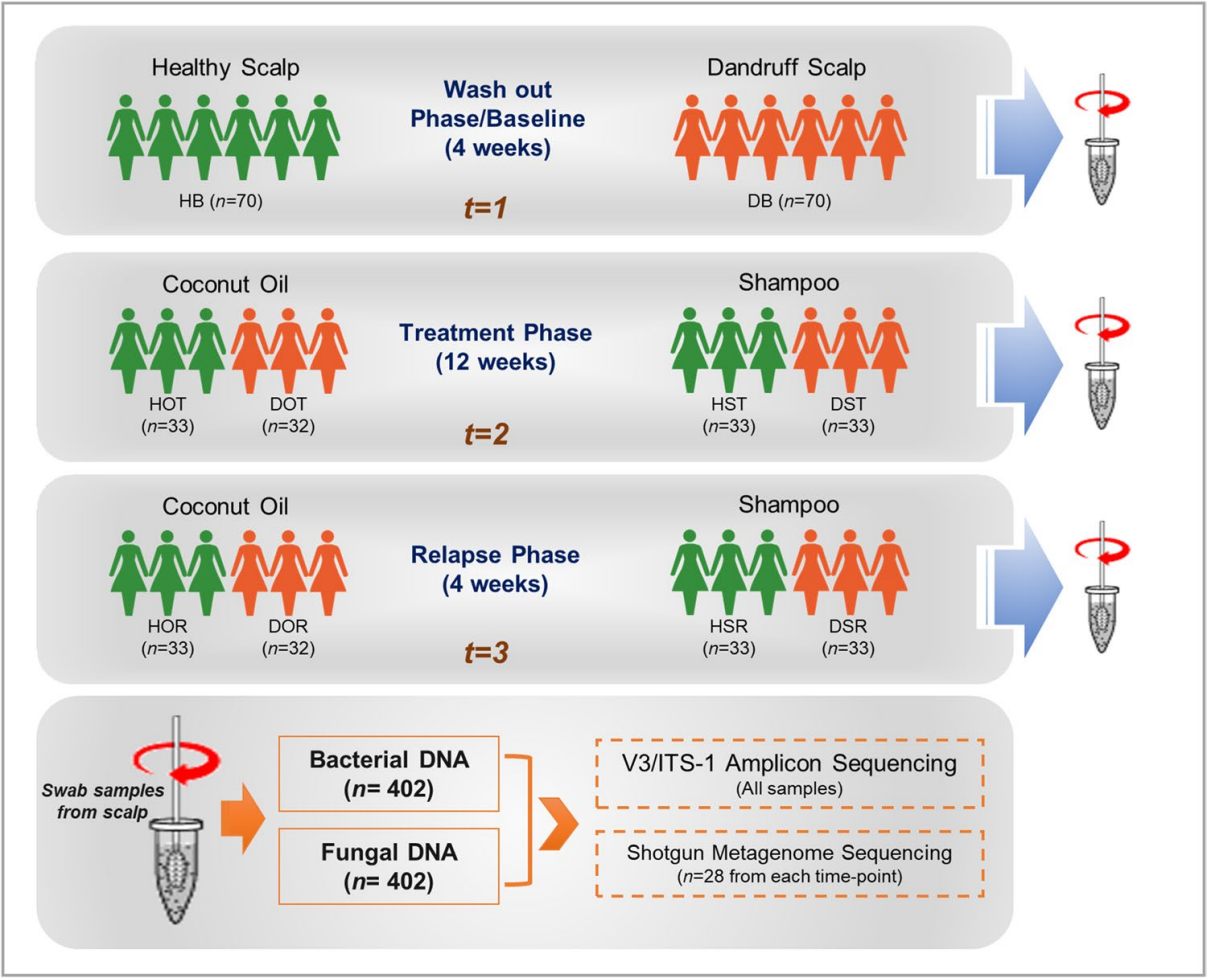
Study Design. Swab samples were collected at three phases, baseline (*t* = 1), treatment phase (*t* = 2) and relapse phase (*t* = 3) from healthy and dandruff scalps. Bacterial and fungal DNA was extracted from the collected swab samples, and amplicon (bacterial 16S rRNA V3 and fungal ITS1 region) and shotgun metagenomic sequencing were performed to carry out the taxonomic and functional analysis. In the figure, H = healthy scalp, D = dandruff scalp, O = oil-treatment, S = shampoo-treatment, and B, T and R = the three phases or time-points i.e. Baseline, Treatment and Relapse phase, and *n* = number of subjects in each group.

Recently, we reported the functional role of scalp microbiome (bacterial and fungal) in a cohort of 140 Indian individuals consisting of 70 individuals with healthy and 70 with dandruff scalp^4^. In the current study, we have used the same cohort to perform a time-course study to understand the impact of coconut oil application on the scalp. Amplicon and shotgun metagenomic analysis revealed the major microbial species and their functional pathways in the healthy and dandruff scalp microbiome at the baseline. From the amplicon analysis, the core scalp microbiome was defined as the taxonomic groups with ≥ 1% abundance in at least 80% of the samples, which represented the stable and consistent microbial population in the microbiome associated with the scalp environment. To examine the effect of coconut oil (O) on the scalp microbiome, the changes in the relative abundance of the core microbiome and their associated functional pathways were analysed and compared with a ‘neutral shampoo’ (S) after 12-weeks of treatment phase (T) followed by four weeks of relapse phase (R). The study design is presented in Fig. 1 and details on the nomenclature of groups and samples is provided in supplementary methods.

### Taxonomic variations in the fungal microbiome after the treatment and relapse phases

Taxonomic analysis of the baseline microflora showed the alpha-diversity of the fungal population to be significantly lower (*p* ≤ 0.001) in the healthy scalp (HB: Healthy scalp Baseline) compared to the dandruff scalp (DB: Dandruff scalp Baseline) (Fig. S1a). A high abundance of *M. globosa* (*p* ≤ 0.0001) was observed in HB (16.23%) compared to DB (6.41%) (Fig. S1b–d). A strikingly high proportion of OTUs corresponding to uncharacterized *Malassezia* spp. was observed. One of these OTUs belonged to uncultured species of *Malassezia* (> 95% identity with Uncultured Malassezia, Genbank ID—KC785585.1) and others belonged to unknown *Malassezia* species (also at > 95% identity), of which, six OTUs (sequences provided in Supplementary Text) showed an identity of ≥ 85% with *M. restricta*, and were assigned to a subgroup of *Malassezia* (i.e. species close to *M. restricta*). Therefore, the uncharacterized Malassezia sequences formed three subgroups: (1) uncultured *Malassezia*, (2) *Malassezia* sp., and (3) species close to *M. restricta*, as described previously^4^. The uncultured *Malassezia* was significantly abundant (*p* ≤ 0.0001) in DB (25.26%) compared to HB (14.44%). *Malassezia* sp. and species close to *M. restricta* were also significantly (*p* ≤ 0.01) higher in DB compared to HB (Fig. S1d).

The alpha-diversity (Shannon index and number of observed species) of the fungal microbiome increased significantly (*p* ≤ 0.0001) after oil treatment (HOT) and after-shampoo application (HST) in the healthy scalp compared to the baseline (HB) (Fig. S1a). It also showed a significant increase (*p* ≤ 0.0001) in oil-treated healthy scalp at the relapse phase (HOR) as compared to baseline (HB) and treatment phase (HOT). However, there was no significant effect seen on the alpha-diversity of the dandruff groups.

**Figure 2:**
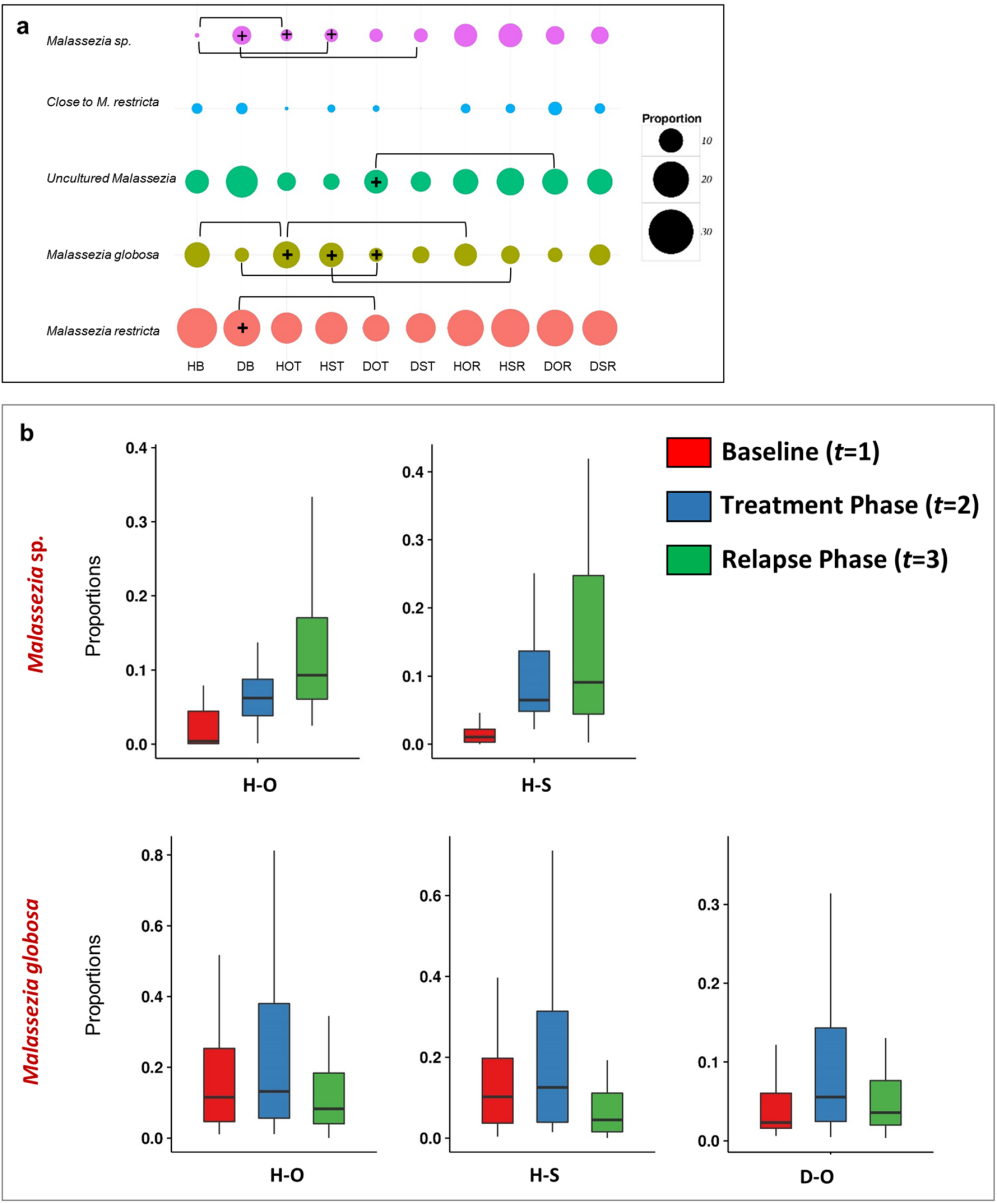
Comparison of fungal population at the three phases. (**a**) Bubble plots representing the top five fungal species across all the groups. The bubble size indicates mean relative abundance of species within each group. Square brackets indicate the groups between which a significant difference in the species abundance was observed (*p* ≤ 0.05, Wilcoxon test, + indicates the group with the higher abundance among the two). (**b**) Core fungal species showing significant variations across the three phases (FDR adjusted *p* ≤ 0.05, repeated measures ANOVA).

Proportions of *M. restricta* and *M. globosa* along with the *Malassezia* subgroups (1, 2 and 3), constituting the fungal core of the scalp, were examined in the three phases. After oil-treatment, the abundance of *M. restricta* reduced significantly (*p* = 0.03) in DOT (26.55%) compared to DB (33%) (Fig. 2a and Fig. S2a–e). The abundance of *M. globosa* increased significantly in both healthy (HOT = 23.23%, *p* = 0.02) and dandruff scalp (DOT = 9.74%, *p* ≤ 0.001) compared to the baseline, i.e. HB (16.23%) and DB (6.41%), respectively. It also increased significantly (*p* ≤ 0.05) in DST (14.32%) compared to DB (6.41%), while there was no significant difference between HST and HB. The abundance of *Malassezia* sp. increased significantly (*p* ≤ 0.0001) in HOT (8.86%) and HST (10%) compared to HB (2.37%). It also increased significantly (*p* ≤ 0.05) in DST (12.28%) compared to DB (10.12%). Additionally, the ratio of *M. restricta* to *M. globosa*, which was observed to be significantly higher (*p* = 0.004) in the dandruff scalp compared to the healthy scalp, decreased significantly (*p* = 0.001) in DOT compared to DB (Fig. S1e).

Group-wise analysis using repeated measures ANOVA confirmed the significant variations (FDR adj. *p* ≤ 0.05) in *M. globosa* after oil-treatment in both healthy and dandruff scalp, and after shampoo-application in the healthy scalp (Fig. 2b and Table S1a). A similar trend was shown by *Malassezia* sp. in oil and shampoo-treated healthy scalp. The abundance of *M. globosa* decreased significantly (*p* ≤ 0.01) in HOR (13.41%) and HSR (9.44%) compared to HOT (23.23%) and HST (19.32%), respectively (Fig. S3a–e). A significant decrease (*p* = 0.03) in the abundance of uncultured *Malassezia* was also observed in DOR (18.37%) compared to DOT (23.60%) (Fig. 2a). These results suggest that the effect of oil-treatment on the changes in the fungal community was not sustained for dandruff group after the relapse phase, in contrast to the healthy group.

### Taxonomic variations in the bacterial microbiome after the treatment and relapse phases

There was no significant difference observed in the alpha-diversity of the bacterial microbiome between healthy (HB) and dandruff (DB) group at the baseline (Fig. S4a). The weighted UniFrac distances does not show a significant difference between healthy (HB) and dandruff (DB) group at the baseline (Fig. S4b). At the species level, the abundance of *S. epidermidis* was observed to be significantly higher (*p* = 0.0002) in dandruff (28.11%) than in healthy (14.83%) scalp (Fig. S4c–e). The abundance of *C. acnes* did not vary significantly (*p* ≥ 0.05) between dandruff (30.83%) and healthy (24.42%) scalp.

After the treatment phase, there was a significant increase (*p* ≤ 0.01) in the bacterial diversity (Shannon index) in the healthy scalp after oil-treatment (HOT) and not with shampoo-application (HST) compared to baseline (Fig. S4a). The weighted UniFrac distances showed a significant decrease in within-sample distances in HOT compared to HB, while it was not significant for DOT compared to DB (Fig. S4b). Interestingly, oil-treated groups (HOT and DOT) showed lower UniFrac distances compared to the shampoo-treated group (HST and DST respectively), suggesting a distinct effect of coconut oil on the scalp bacterial communities.

**Figure 3:**
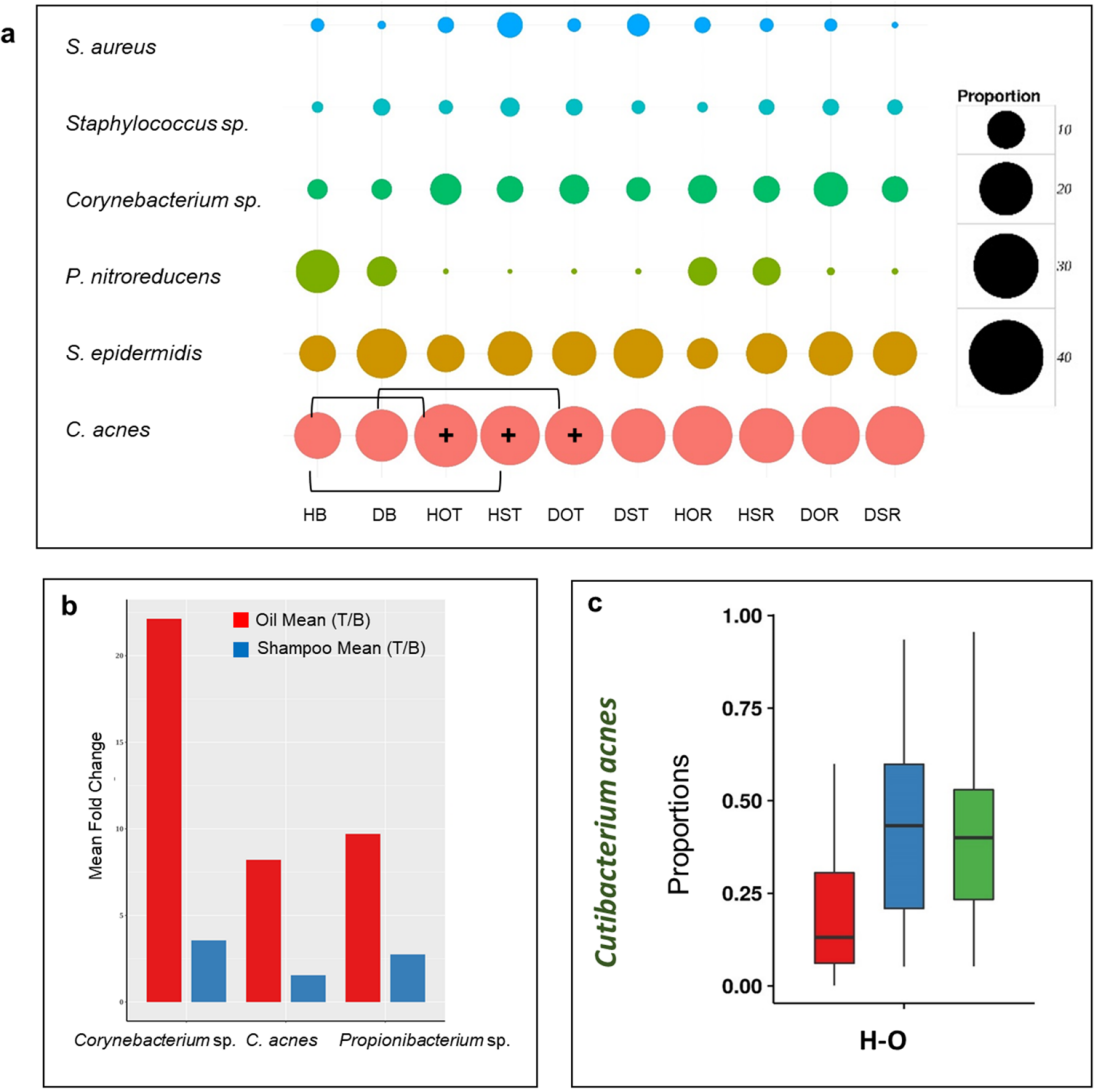
Comparison of bacterial population at the three phases. (**a**) Bubble plots representing the top five bacterial species across all the groups. The bubble size indicates mean relative abundance of species within each group. Square brackets indicate the groups between which a significant difference in the species abundance was observed (*p* ≤ 0.05, Wilcoxon test, + indicates the group with the higher abundance among the two). (**b**) Significant fold-change differences (*p* ≤ 0.05) observed in bacterial species abundance between oil-treatment and shampoo-application. No significant difference was observed in the fungal species between the two treatment groups. (**c**) Core bacterial species showing significant variations across the three phases (FDR adjusted *p* ≤ 0.05, repeated measures ANOVA).

At the taxonomic level, we have focussed on the variations in the abundance of the two main species *C. acnes* and *S. epidermidis* that constitute the core microbiome. The proportion of *C. acnes* increased significantly (*p* ≤ 0.0001) in HOT (45.29%) compared to HB (24.42%) (Fig. 3a and Fig. S5a–e). Although less than HOT, it also showed a significant increase (*p* ≤ 0.0001) in HST (40.09%) compared to HB. The ratio of *C. acnes* to *S. epidermidis*, which was significantly higher (*p* = 0.01) in the healthy scalp compared to the dandruff scalp, was significantly higher (*p* = 0.008) in HOT compared to HB (Fig. S5f). Significant fold-change differences were observed in the abundance of bacterial species between oil-treatment and shampoo-application (Fig. 3b). Interestingly, *C. acnes* and *Propionibacterium* sp. showed significant (*p* ≤ 0.05) fold-changes after oil-treatment compared to shampoo-application. Fold change analysis was applied to both bacterial and fungal population, however the results were found to be significant (*p* ≤ 0.05) only for the bacterial microbiome. We did not find any fungal species to show significant fold-changes between the groups and hence, the results only from the bacterial microbiome are included here. Among the other species, *Corynebacterium* sp., showed the maximum level of fold-change (from < 5 to > 20 times, *p* ≤ 0.05) after oil-treatment. Repeated measures ANOVA (FDR adj. *p* ≤ 0.05) confirmed an increase in the abundance of *C. acnes* in healthy subjects after oil-treatment (Fig. 3c, Table S1b). Changes in the proportion of *C. acnes* and *S. epidermidis* were retained (*p* ≥ 0.05) after the relapse phase (Fig. 3c and Fig. S6). These results suggest an apparent beneficial effect of coconut oil on the core bacterial species after 12 weeks treatment, which appears to be retained at the relapse phase.

### Correlation of microbial species with host physiological parameters

Dandruff scalp is characterized by in increased TEWL indicating an altered barrier function, and a few studies have described the variation in the host-associated physiological parameters in the healthy and dandruff scalp^8,41^. Therefore, a systematic investigation of the host physiological parameters was carried out at all the three phases of the study and correlated with the scalp microbiome (Table S2).

**Figure 4:**
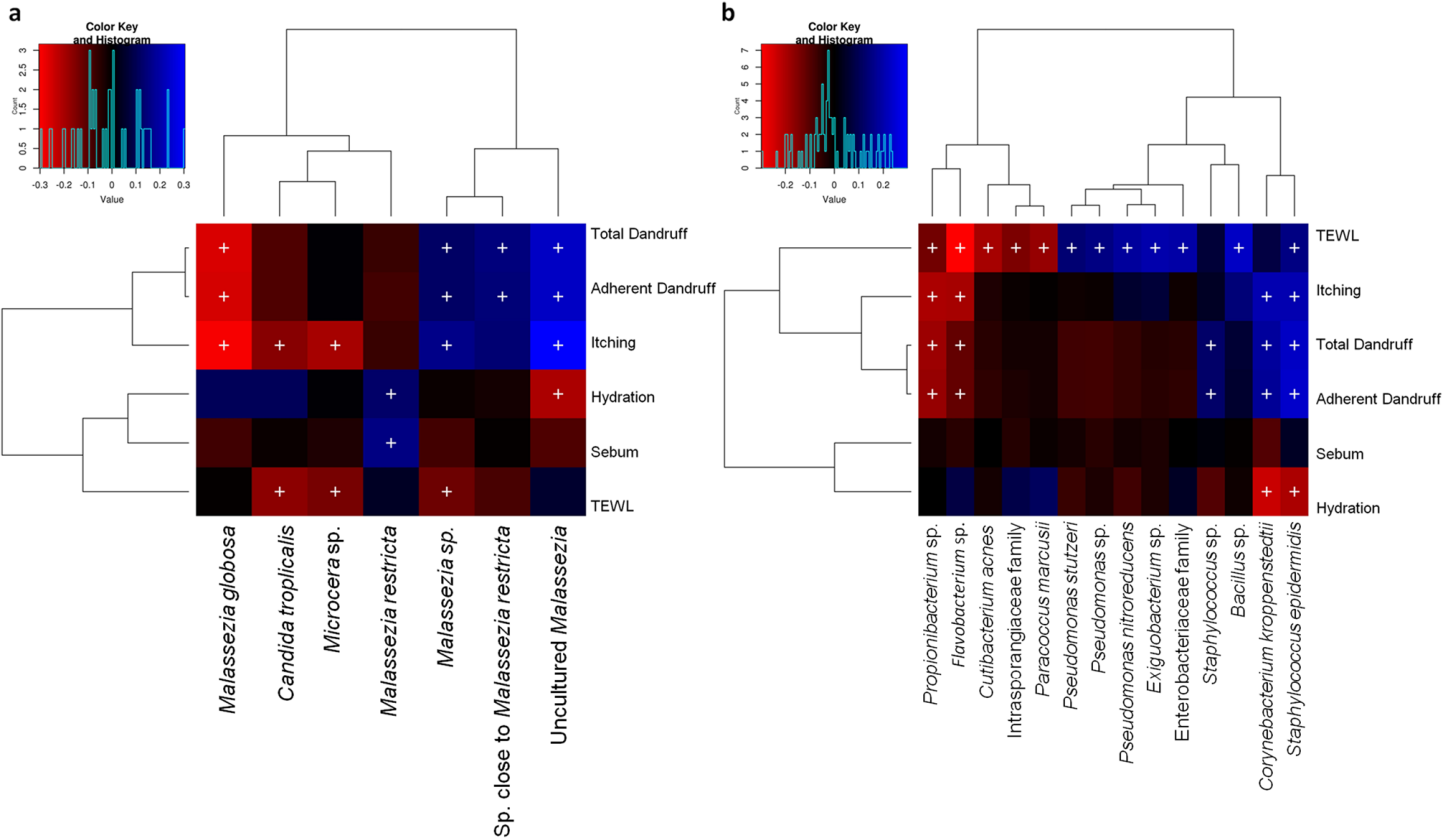
Spearman’s correlation between microbial taxa and host physiological parameters. (**a**) Fungal and (**b**) bacterial taxa showing significant correlations (+ , FDR adjusted *p* ≤ 0.05) with any of the parameters are plotted as a heatmap.

The three *Malassezia* subgroups showed significant positive correlation (FDR adj. *p* ≤ 0.05) with dandruff scores and itching, whereas, *M. globosa* was negatively correlated with these parameters (Fig. 4a). The taxa corresponding to unknown *Malassezia* sp. showed a significant negative correlation with TEWL. Uncultured *Malassezia* showed a negative correlation with hydration, and *M. restricta* showed a positive correlation with sebum level and hydration.

Among the bacterial population, *S. epidermidis* displayed a significant positive correlation (FDR adj. *p* ≤ 0.05) with dandruff scores, TEWL and itching. However, *Propionibacterium* sp. correlated negatively with these parameters, and *C. acnes* showed a significant negative correlation with TEWL (Fig. 4b). Although being low in abundance, *Flavobacterium* sp. and *C. kroppenstedtii* showed a pattern similar to *Propionibacterium* sp. and *S. epidermidis,* respectively.

The host physiological parameters were also compared in the oil and shampoo-treated groups across the three phases using repeated measures ANOVA. The results showed a reduction in TEWL and dandruff scores in both healthy and dandruff groups after the coconut oil treatment phase (Table S1c). It is to be noted that the results for coconut oil treatment are more concordant when studied at taxonomic level compared to the host physiological parameters, which are influenced by many other factors beyond the scope of this study.

### Functional variations in the scalp microbiome

**Figure 5:**
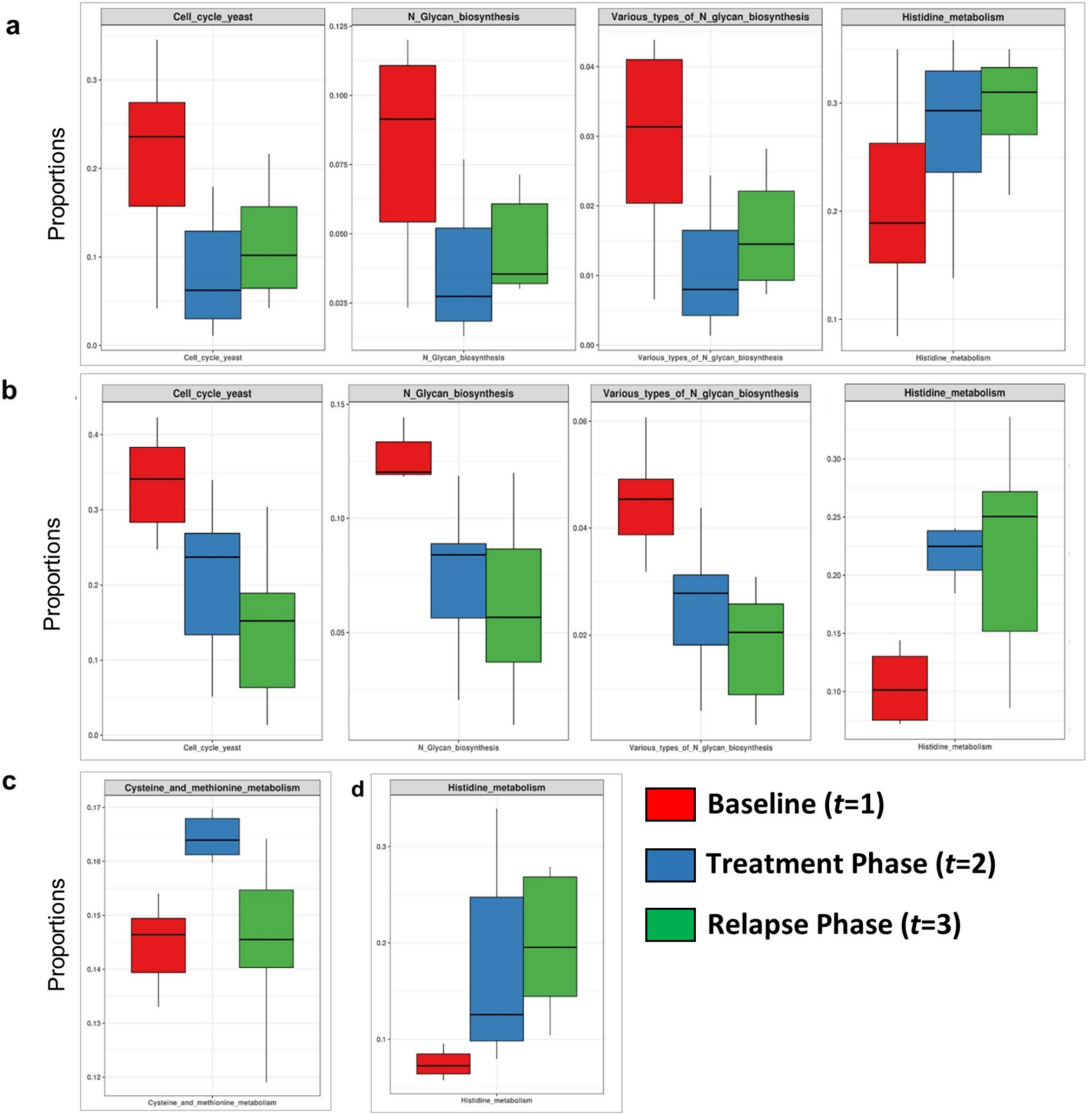
Functional analysis of fungal microbiome. The proportions of fungal pathways showing significant variations across all the three phases in the following groups (FDR adjusted p ≤ 0.05, repeated measures ANOVA) are shown in the box plots: (**a**) Healthy oil-treated group, (**b**) dandruff oil-treated group, (**c**) healthy shampoo-treated group and (**d**) dandruff shampoo-treated group.

The shotgun metagenomic analysis showed several fungal KEGG pathways to vary significantly (*p* ≤ 0.05, Wilcoxon test) between the healthy and dandruff scalp at the baseline as reported earlier (Fig. S7a)^4^. In brief, the amino acid metabolism pathways (histidine, cysteine and methionine metabolism) and lipoic acid metabolism pathway were more abundant in healthy scalp than in dandruff scalp. Further, pathways for N-glycan biosynthesis, which are implicated in cell-host interaction, were enriched in the dandruff scalp^42^. Several fungal pathways also showed significant variations in their proportions after the treatment phase, which were statistically tested at all three phases using repeated measures ANOVA (Table S1d; Fig. 5). After oil-treatment, histidine metabolism pathway showed a significant increase in both healthy and dandruff groups, however, it was also observed in the shampoo group suggesting that it is not linked to the oil application. In contrast, the proportion of N-glycan biosynthesis and cell cycle pathways decreased in both healthy and dandruff scalps in the oil treated group and not in the shampoo group compared to baseline (Fig. 5). This effect was not sustained at the relapse phase, since there was a significant increase at the relapse phase (*t* = 3) compared to the treatment phase (*t* = 2). However, the initial baseline levels were not recovered.

**Figure 6:**
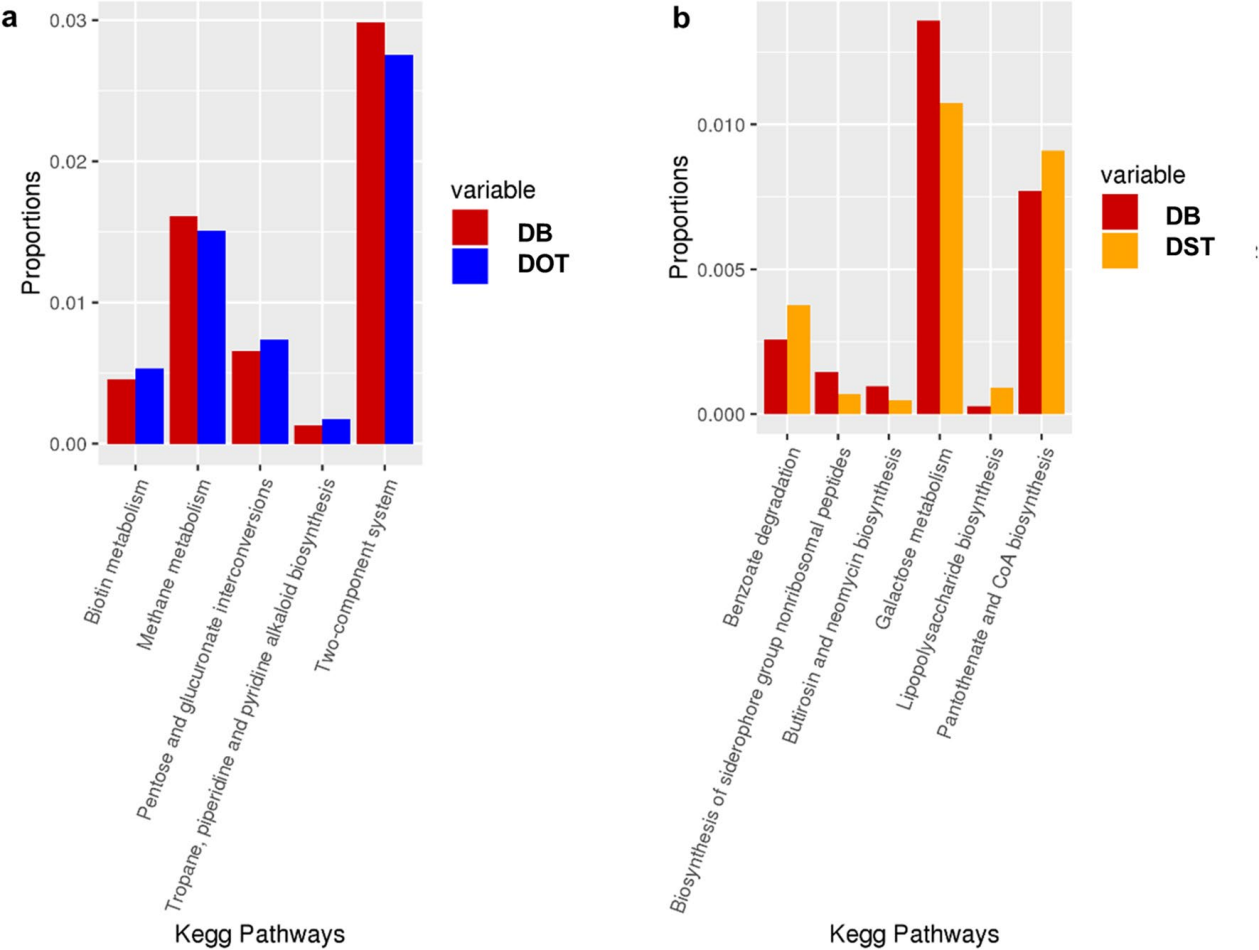
Functional analysis of bacterial microbiome. Differentially abundant bacterial KEGG pathways after (**a**) oil-treatment and (**b**) shampoo application in the dandruff group (which showed p ≤ 0.05, Wilcoxon test) are shown in the bar graphs. None of the pathways showed significant differences in the healthy group.

**Figure 7:**
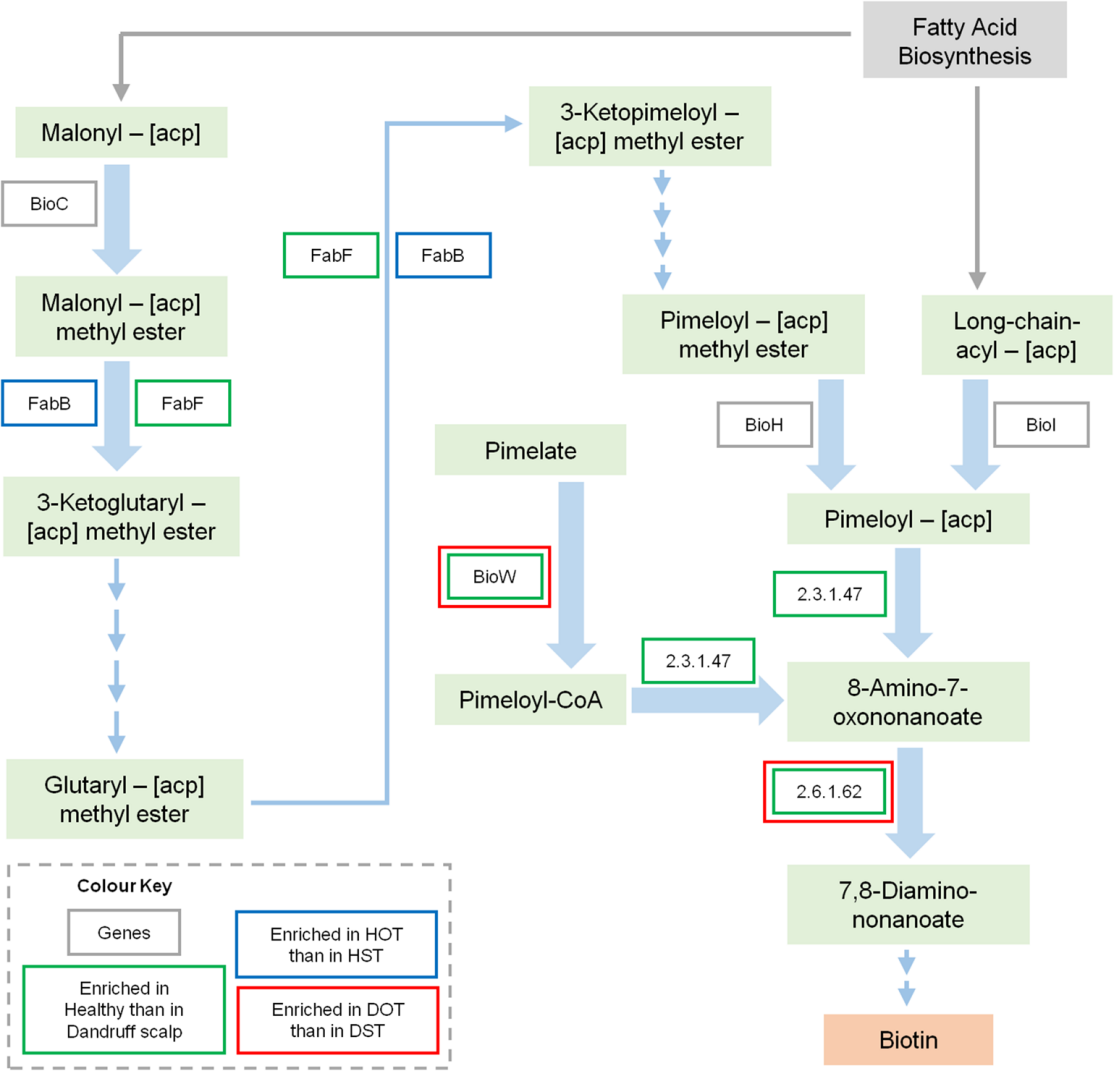
Schematic representation of biotin metabolism pathway describing the significantly enriched KOs (*p* ≤ 0.05) in the dataset.

Functional analysis of the bacterial microbiome showed the pathways related to vitamins and cofactors (biotin, porphyrin and chlorophyll, vitamin-B6, nicotinate and nicotinamide metabolism, ubiquinone and other terpenoid-quinone biosynthesis), and amino acids (alanine, aspartate, arginine, glutamate and proline and lysine metabolism and biosynthesis) to be significantly higher in healthy scalp than dandruff scalp at the baseline, as reported earlier (Fig. S7b)^4^. Results from the previous study have shown these pathways to be positively correlated with *C*. *acnes*, suggesting it to be the major contributor for biotin and other B-vitamins on the scalp surface^4^. Bacterial KEGG pathways showed significant variations after oil-treatment compared to shampoo-application in the dandruff scalp (Fig. 6). The biotin metabolism pathway showed a substantial increase in DOT compared to DB. The variations in pathway abundance were also tested statistically at the three phases using repeated measures ANOVA (Table S1e). The abundance of KOs related to biotin metabolism and biotin transport pathways also increased after oil-treatment compared to shampoo-application in the healthy and dandruff scalp (Fig. 7, Fig. S7c and d).

## Discussion

This study reports the effect of topical application of coconut oil on the scalp microbiome in a cohort of 140 Indian women using high-throughput sequencing and computational analysis. Since, bacteria and fungi are the major scalp microbial species, we examined both using bacterial 16S rRNA and fungal ITS1 amplicon sequencing, respectively, followed by the shotgun metagenomic sequencing, which helped to understand the impact of coconut oil on the scalp microbiome.

Coconut oil is commonly used in several parts of the world to maintain scalp health and to moisturise the skin in addition to repair hair damage, through a direct or indirect mode of action^29,31^. Supporting studies have demonstrated the inhibitory role of coconut oil and its major component, lauric acid, on the growth and invasion of dermatophytes on the skin more efficiently than other hair oils commonly used in India such as mustard oil, cantharidine oil, amla oil, etc.^32–34^. It is also known to decrease the TEWL on long-term application on the skin surface, and increases the hydration levels^36^, which could play an important role in shaping the microbiome of the scalp surface. Thus, it seems reasonable to speculate a potent effect of coconut oil application on the scalp physiology, which in turn modifies the scalp microbiome^4,8^.

In our previous study of the scalp microbiome in the Indian cohort, we have obtained the baseline microbial composition of both fungal and bacterial communities, which has been reported and reanalysed for the current study^4^. The determination of baseline microflora helped to elucidate the potential beneficial role of microbiome by comparing their compositional changes after the treatment phase. *Cutibacterium acnes* and *Staphylococcus epidermidis* emerged as major bacterial colonizers, and *Malassezia restricta* and *Malassezia globosa* as the major fungal colonisers. Similar association of these species with the healthy and dandruff scalp has also been observed in other studies from different population such as, from France, China, Brazil, etc.^5–8^. A noteworthy observation at the baseline was the high abundance of uncharacterised *Malassezia* sp. in the dandruff scalp compared to the healthy scalp, and its significant positive correlation with dandruff-associated parameters. A high proportion of uncultured *Malassezia* (> 37% of the total fungal population) has also been observed recently in the Brazilian population^7^. The results also showed *M. globosa* to be negatively correlated with dandruff score and itching, and *S. epidermidis* and uncharacterised *Malassezia* sp. to be positively correlated with these parameters. Similar variation in the proportion of *M. globosa* was also reported in a Chinese cohort using the Illumina sequencing technology^8^. However, a few other studies have reported contrasting results, which could be a result of lower cohort size, differences in the country of origin of populations and usage of conventional sequencing technologies^5,7,9,43^.

The above taxonomic results were confirmed in this study by recording the host physiological parameters at all the three time-points. The results also showed the beneficial role of bacterial scalp microbiome in supplying essential vitamins and amino acids to the host as observed in the previous study, where a positive correlation of *C. acnes* was also observed with the pathways related to the metabolism of biotin and other B-vitamins that are essential for maintaining a healthy scalp^4^.

The time-course study revealed an apparent beneficial effect of coconut oil application on the fungal scalp microbiome in enhancing the core fungal species associated with a healthy microflora. The abundance of *M. globosa*, which was correlated with the healthy scalp, increased after the application of coconut oil in the healthy and dandruff scalp. The abundance of *M. restricta* reduced significantly in the dandruff scalp after oil-treatment as compared to the baseline. Further, a significant decrease in the ratio of *M. restricta* to *M. globosa* was observed in dandruff subjects after the application of coconut oil. This ratio was similar to the baseline healthy composition reported in this cohort and other populations, which points towards a probable role of coconut oil in maintaining a healthy fungal scalp microbiome^7^. The pathways related to pathogenesis, survival and adhesion (N-glycan biosynthesis and cell cycle pathways) showed a significant reduction after the application of coconut oil, suggesting its role in lowering the abundance of fungal pathogenic species. The effect of coconut oil was not sustained for dandruff group, mainly in the fungal community. This observation could be explained by a limited effect of the oil on the fungal microbiome in the dandruff scalp. The fungal taxa found on the dandruff scalp were significantly different compared to healthy scalp and could be more resistant to lauric acid.

The beneficial effects of coconut oil were more prominently evident on the bacterial microbiome compared to the fungal microbiome. It was observed that *C. acnes* and *Propionibacterium* sp., which are reportedly associated with a healthy scalp, showed a significant increase after oil-treatment in both the healthy and dandruff scalp^4–7^. Further, the ratio of *C. acnes* to *S. epidermidis* was also notably increased after the oil-treatment. These results suggest that oil helps in enhancing the beneficial bacterial species in both healthy and dandruff scalp. This was further confirmed by the significant increase in the biotin metabolism pathway after oil-treatment, which was majorly contributed by *C. acnes* and *Propionibacterium* sp. Biotin and other B-vitamins are crucial precursors for different enzymes required for vital biochemical reactions in the living cells, and are also essential for a healthy skin and scalp surface^44^. Biotin is also reported to reduce cellular inflammation and improve skin barrier quality, scalp health and hair growth^35,36^. Since, humans and other mammals cannot synthesize biotin, they obtain it through exogenous sources by import mechanism of the Na-dependent multivitamin transporters (SMVT) and biotin-specific transport components^45,46^. Thus, it is tempting to speculate the beneficial effect of coconut oil on the scalp microbiome primarily via creating physiological conditions that favour the beneficial microbial community involved in the biosynthesis of nutrients essential for scalp nutrition.

This study demonstrates a positive effect of coconut oil on the scalp microbial communities and their functional potential. We could speculate that microbiome changes are the first step towards the restoration of a healthy scalp that will lead to perceptible benefits to host much later than the time-lines included in this study, and thus provide a long-term benefit compared to short-term benefit as observed in the case of the neutral shampoo. Another significant outcome of the study is that beyond antifungal agents, other approaches could be considered for dandruff treatment targeting both fungal and bacterial microbiome. However, further studies are needed to understand the underlying mechanisms and nutritional significance of coconut oil for the scalp microbiome. A pre-emptive approach aimed at reducing the susceptibility to dandruff by maintaining/reinstating a healthy scalp microbiome, in addition to improving scalp barrier functions, seems a novel opportunity to achieve long-lasting effects.

## Materials and methods

### Ethics approval and consent to participate

The research protocol was approved by the Independent Ethics Committee for Evaluation of Protocols for Clinical Research, CLINICOM, Bengaluru, India (Study number ARC/COSB/1444) and was conducted in accordance with the principles expressed in the World Medical Association Declaration of Helsinki. A written informed consent was obtained from all subjects prior to any study-related procedures.

### Subject recruitment and study design

The study was carried out on a previously reported cohort of 140 Indian women^4^. Firstly, 184 female volunteers were screened for the study, out of which 70 individuals with healthy and 70 with dandruff scalp (aged between 20–45 years, mean age 34.6) were enrolled with the association of MS Clinical research (Bengaluru, India), who had used coconut oil occasionally in the past one year. The volunteers were non-smokers, free from any cutaneous diseases, did not use anti-hair-loss treatment at least for three months prior to sampling, did not use anti-dandruff shampoos and hair-related products (such as for bleaching, straightening, dyeing, permanent waving, etc.) on scalp and hair for at least three weeks prior to sampling, and did not consume antibiotics or apply systemic antifungals for one month prior to sampling.

### Dandruff grading and clinical evaluation of scalp physiological parameters

Dandruff level was scored according to a grading scale as previously described^5^. Scalp physiological parameters were measured using appropriate devices and protocols. Sebumeter (SM815, Courage & Khazaka, Germany) was used to measure the sebum level of the scalp following manufacturer’s instructions. VapoMeter (Delfin Technologies, Finland) was used to measure the TEWL (trans-epidermal water loss) that measures the integrity of barrier function of the scalp by following the manufacturer’s instructions^47^. All the measurements were performed in triplicates and values presenting coefficient of variation < 15% were considered as relevant. Corneometer (CK Electronic GmbH, Germany) measurement was performed on the scalp surface to check the hydration levels following the manufacturer’s instructions. The above measurements were performed on a shaved trimmed mini-area before the oil-treatment and shampoo wash at the investigational site by a dedicated operator. Following clinical or physiological parameters were recorded for each subject across the three phases: adherent dandruff score (ADS), total dandruff score (TDS) i.e. adherent and non-adherent, hydration, sebum level, TEWL, erythema and itching (Table S2).

### Treatment

The volunteers were asked to use a bland or neutral shampoo (L’Oréal India Pvt. Ltd.) two times a week for a period of four weeks prior to the beginning of the study to standardize the scalp condition (baseline, Day-0 or *t* = 1). Four subjects from the healthy group and five from the dandruff group did not continue after the baseline sampling, and therefore, the remaining 66 subjects with healthy scalp and 65 with dandruff scalp were followed-up throughout the course of the study. During the treatment phase (Day-84 or *t* = 2), 32 subjects with dandruff scalp and 33 with healthy scalp received controlled oil-treatment twice a week for 12 weeks at the center (MS Clinical Research, Bengaluru, India). The treatment consisted of 20 min of scalp massage with 10 ml of pure coconut oil (100% refined coconut oil, Cargill, India) followed by two hours leave-on, and then a bland shampoo wash (20 ml). The remaining 33 subjects with dandruff scalp and 33 with healthy scalp were subjected to only the shampoo wash twice a week at the center (MS Clinical research, Bengaluru, India) for 12 weeks with no application of coconut oil. During the relapse phase (Day-112 or *t* = 3), all the 131 subjects received a topical application of only the bland shampoo on the scalp twice a week for four weeks. Subjects were advised not to use any hair or scalp products other than the study products.

### Sampling of the scalp microbiome

The volunteers were asked not to perform scalp wash for two days prior to the sampling procedure. Samples from the scalp (vertex or crown of the head) were obtained at the baseline (*t* = 1), at the end of 12 weeks of treatment phase (*t* = 2) and after the relapse phase (*t* = 3). Sampling was conducted as previously described with minor modifications^5^. A sterile cotton swab soaked in a solution containing collection solution (0.15 M NaCl and 0.1% Tween 20) was rubbed onto the scalp surface (between the hairs) under a zig-zag pattern, to cover a total surface of 4 cm^2^ in a non-overlapping manner. At the end of the procedure, the head of each swab was cut from the handle and placed into a tube containing 5 ml of collection buffer. As described previously^5^, swabs were stored at 4 °C and processed for DNA isolation within 24 h. In addition, a few sterile cotton swabs (as negative controls) were cut from the handle and placed in the collection buffer, and further processed using identical procedure.

### High-throughput sequencing DNA extraction

The DNA extraction method was validated, as described above^4^. Since, the DNA extraction strategy can influence the microbiota community profiling, the DNA extraction method was developed using different bacterial fungal species and ensured that maximum quantity of DNA was recovered by the optimized protocols.

Two identical samples of 2 ml each were generated from one collection tube. The microbial cell suspension from each tube was pelleted by centrifugation at 10,000 g for 30 min, at 4 °C. For bacterial DNA extraction, the cells were re-suspended in 180 ml of lysis buffer (20 mM Tris–HCl, 2 mM EDTA, 1.2% TritonX100 (w/v); pH 8.0) and incubated for 30 min at 37 °C. Further 25 µl of proteinase K and 200 µl buffer AL (Qiagen, MD, USA) were added to the mixture and incubated for 30 min at 56 °C.

For fungal DNA extraction, the cells were re-suspended in 600 ml of lysis buffer (1 M Sorbitol, 100 mM EDTA, 14 mM β-mercaptoethanol) and incubated with Zymolyase-T20 (200 U) for 30 min at 30 °C. The resulting spheroplasts were centrifuged for 10 min at 300 × g and the supernatant was discarded. The spheroplasts were re-suspended in 180 µl of Buffer ATL and incubated with 20 µl of proteinase K (Qiagen, MD, USA) for 15 min at 56 °C.

The remaining steps were performed according to the manufacturer’s protocol and the extracted DNA was stored at -20 °C. DNA concentration was measured using Qubit ds DNA HS kit on Qubit 2.0 fluorometer (Life technologies, Carlsbard, CA, USA).

### PCR amplification and high-throughput sequencing

The PCR amplification and high-throughput sequencing was performed as explained in a previous study^4^. Equal concentration of bacterial and fungal DNA was used (~ 1 ng) for PCR amplification of 16S rRNA V3 hypervariable region and ITS1 region, respectively (see supplementary methods for details on primers and protocol used). Four bacterial samples and two fungal samples did not show any amplification, and therefore were not included in the study. After evaluating the amplified products on 2% w/v agarose gel, the products were purified using Ampure XP kit (Beckman Coulter, Brea, CA USA). Amplicon libraries were prepared using primers for V3 and ITS1 regions by following the Illumina 16S metagenomic library preparation guide. The metagenomic libraries were prepared using Illumina Nextera XT sample preparation kit (Illumina Inc., USA).

Based on the minimal DNA concentration (> 0.2 ng/µl) required to carry out the library preparation for shotgun sequencing, 14 subjects with healthy scalp and 14 with dandruff scalp were selected from each time-point for shotgun metagenome sequencing of their bacterial and fungal DNA. Thus, a total of 398 bacterial and 400 fungal amplicon samples, and 84 bacterial and 84 fungal metagenomic samples were sequenced in this study (Table S2).

Both the amplicon and shotgun metagenome libraries were evaluated on 2100 Bioanalyzer using DNA1000 kit for amplicon, and High Sensitivity DNA kit for metagenome (Agilent Technologies, Santa Clara, CA, USA) to estimate the library size. The libraries were further quantified on a Qubit 2.0 fluorometer using Qubit dsDNA HS kit (Life technologies, USA) and by qPCR using KAPA SYBR FAST qPCR Master mix and Illumina standards and primer premix (KAPA Biosystems, Wilmington, MA, USA) following the Illumina suggested protocol. Libraries in equal concentrations were loaded on Illumina NextSeq 500 platform using NextSeq 500/550 v2 sequencing reagent kit (Illumina Inc., USA) and 150 bp paired-end sequencing was performed for both types of libraries at the Next-Generation Sequencing (NGS) Facility, IISER Bhopal, India.

### Assignment of bacterial 16S rRNA (V3) and fungal ITS1 amplicon reads

The raw sequence data was subjected to quality trimming and ambiguity filtering using NGSQC toolkit and the paired-end reads were assembled for each amplicon sequence using FLASH^48,49^. Quality filtration of fastq reads was carried out using NGSQC toolkit and all the reads with 80% of bases ≥ Q30 quality scores were selected for further analysis. Primers from the reads were removed using Cutadapt^50^ and reads without the primer sequences were discarded. Clustering was carried out using closed-reference OTU picking and de novo OTU picking protocol of QIIME v1.9^51^ at ≥ 97% identity. The custom ITS1 database prepared previously^4^ and Greengenes database v13_5 were used as a reference for fungal and bacterial taxonomic assignment, respectively^52^.

α-diversity was calculated using the Shannon index metrics and observed species after rarefying from 100 sequences at a step size of 6,000 for V3 as well as for ITS1 amplicons using QIIME v1.9. Pielou’s evenness was calculated to identify the distribution of species with respect to their proportion in each sample groups using the R-package^53^. Weighted UniFrac distances were measured for the bacterial population, and not for fungal samples, due to the highly variable nature of ITS1 sequences, which makes them difficult to interpret informative and meaningful phylogenetic information^54,55^.

For the taxonomic assignment of de novo OTUs, sequences were aligned against the respective databases using BLAT, and the assignment was performed using Lowest Common Ancestor (LCA) algorithm^52,56^. The negative control samples showed a high abundance of fungal genus *Pachysolen* and bacterial genus *Actinotalea*, which are commonly found in environments such as air and soil, and not associated with the skin or scalp^57,58^. Hence, the OTUs from these genera were excluded from the analysis.

### Shotgun metagenomic data analysis

Metagenomic reads with 60% bases above Q25 were considered for the analysis^49^. For fungal metagenome, the bacterial contaminant reads were removed by alignment against the bacterial reference genomes retrieved from NCBI, and the human contaminant reads were removed using BMTagger^59^. The remaining fungal reads from each sample were assembled independently into contigs using SOAPdenovo at a k-mer size of 75 bp.

For bacterial metagenome, the human and fungal contaminant reads were removed by aligning the sequences using BLAT against human HG19 assembly and custom fungal genome database, respectively^60^. These fungal genes and genomes were downloaded from Aspergillus database, FungiDB (release-30), Fungal Genome Initiative-Broad Institute, Fungi Ensembl, Saccharomyces Genome Database, Candida Genome Database and NCBI to construct a custom fungal genome database^56,61–65^. If any fungal gene sequences were not available in these databases, the gene sequences were extracted from the genome sequences based on the gtf information using SAMtools^66^. The high-quality reads were assembled at a k-mer length of 47 (the k-mer length was estimated using kmerGenie^67^) using SOAPdenovo, and the genes were predicted from the assembled contigs using MetaGeneMark^68,69^.

The fungal contigs with ≥ 300 bp length were selected and fungal genes were predicted from scaffolds using AUGUSTUS^1,68,70^. In order to increase the coverage of genes from fungal genomes found in our dataset, a total of 2,421,207 CDS were added from 303 fungal genomes from Ensembl fungi database. For bacteria, an additional parameter of identity > 50% with ≥ 60% coverage or aligned length > 300 was used. The relative abundance of each KO was calculated by adding up the abundance of genes mapping to the same KO ID, which was then used to calculate the relative abundance of KO ID in each sample. A similar approach was used to calculate the relative abundance of eggNOGs in each sample.

Non-redundant bacterial gene catalogue was generated by using CD-HIT, which consisted of 19,729,749 bacterial genes. A total of 207,763 genes were used as a reference fungal gene set consisting of non-redundant 27,937 genes from assembled metagenomes combined with CDS obtained from Ensembl Fungi genomes database^71^. In total, 587,400 bacterial and 81,395 fungal non-redundant genes were identified in the dataset (see supplementary methods for details on gene quantification).

### Taxonomic assignment of reads from metagenomic data

The fungal reference genomes were retrieved from National Centre for Biotechnology Information (NCBI). The archaeal and bacterial genomes were retrieved from NCBI and Ensembl database. The metagenomic reads were aligned to the reference fungal and bacterial genomes and the mapped reads were considered for further analysis^72,73^.

### KEGG assignment of genes

The KEGG v60 was updated by retrieving new sequences for KO IDs from the KEGG server^74^. The bacterial and fungal genes were annotated by alignment against KEGG and eggNOG v4.0 databases^75–78^. Protein sequences were assigned to eggNOG and KEGG orthologous groups based on the highest scoring hit containing at least one HSP (highest-scoring segment pair) above 60 bits and E-value ≤ 10^–6^ as described previously^4^. In total, 4,064 and 3,532 KO IDs were obtained from the combined fungal and bacterial gene catalogue, respectively. Pathway abundance was calculated by adding the relative abundance of each KO ID belonging to a particular pathway.

### Statistical analysis

The species abundance and KEGG pathway composition were compared between different groups using Wilcoxon test to identify significant (*p* ≤ 0.05) variations. All the comparisons were performed pairwise for each group using R software^79^. In this study, same subjects were sampled at three different phases, therefore, to identify the species and pathways that presented significant (*p* ≤ 0.05) variations across the three phases, repeated measures ANOVA was performed. The species that showed a relative abundance of ≥ 1% in at least 20 samples were considered in this analysis. To identify the significantly varying fold-change in species abundance due to treatment, the fold-changes in species abundance were calculated as *t* = 2/*t* = 1 for each subject and compared using Wilcoxon test.

The Spearman’s Rank Correlation Coefficients with FDR adj. *p*-value were calculated to correlate clinical parameters with species. Species with ≥ 1% proportion in at least 20 samples were selected for the correlation analysis. Correlations were tested across all the phases to obtain the largest set of values for each parameter. Hierarchical clustering algorithm was used for clustering the highly-correlated pathways and species in the samples.

Figures 2b, 3b, 5, 6, S7a and S7b were created using ‘ggplot2’ package in R version 3.0^80^. Figure 4 was created with ‘heatmap2’ function using ‘gplots’ package in R^79^. The Figures S4a and S4b were created using the ‘boxplot’ function from ‘graphics’ package in R^79^.

## Supplementary Information

Supplementary Information 1.

Supplementary Information 2.

Supplementary Information 3.

Supplementary Information 4.

Supplementary Information 5.

Supplementary Information 6.

Supplementary Information 7.

Supplementary Information 8.

Supplementary Information 9.

Supplementary Information 10.

Supplementary Information 11.

## Data Availability

The high-throughput sequence data generated from this study have been deposited under the project number PRJNA415710 in the NCBI BioProject database and will be made publicly available on publication or on request at the time of peer review.
