# Supplementary material for: Longitudinal study of the scalp microbiome suggests coconut oil to enrich healthy scalp commensals
Source: Sci Rep. 2021 Mar 31;11:7220. doi: 10.1038/s41598-021-86454-1 (PMC8012655; doi:10.1038/s41598-021-86454-1)
Supplement: Supplementary file 1 — Supplementary Information 1. [file 41598_2021_86454_MOESM1_ESM.docx]

**Supplementary Methods**

**Title:** Longitudinal study of the scalp microbiome suggests coconut oil to enrich healthy scalp commensals

**Authors:** Rituja Saxena^a†^, Parul Mittal^a†^, Cecile Clavaud^b†^, Darshan B Dhakan^a^, Nita Roy^c^, Lionel Breton^b^, Namita Misra^b,c*^, Vineet K Sharma^a*^

**Affiliation:** ^a^Department of Biological Sciences, Indian Institute of Science Education and Research Bhopal, India, ^b^L’Oréal Research & Innovation, France and ^c^L’Oréal India Pvt. Ltd., India

*Corresponding authors

Vineet K Sharma:

Metagenomics and Systems Biology Laboratory, Academic Building 3, Department of Biological Sciences, Indian Institute of Science Education and Research Bhopal, Bhauri, Bhopal – 462066, India. Tel: +91 755 6691401, email: [vineetks@iiserb.ac.in](mailto:vineetks@iiserb.ac.in)

Namita Misra: [namita.misra@rd.loreal.com](mailto:namita.misra@rd.loreal.com)

†These authors contributed equally to this work

**Nomenclature of samples and groups**

For better comprehension, the nomenclature of all the samples is made as: ‘time-point (*t=*1, 2 or 3) Fungal/Bacterial (F/B)– subject number’. For e.g. 1F-001 (fungal sample from *t*=1 of subject 001), 2B-001 (bacterial sample from *t*=2 of subject 001). For samples sequenced for metagenome, ‘M’ is placed before the sample name, for e.g. M1B-006. Further, the nomenclature of all the groups is made as: ‘condition (H or D) -treatment (O or S) -Phase (B, T or R i.e., Baseline, Treatment of Relapse phase)’. For e.g. HOT (Healthy scalp, Oil-treatment, Treatment phase), DSR (Dandruff scalp, Shampoo-application, Relapse phase).

**PCR amplification of bacterial 16S rRNA V3 region and fungal ITS1 region**

The amplification of bacterial 16S rRNA V3 region was performed using Illumina Nextera XT adapter-ligated eubacterial V3 region-specific primers, 341F and 534R, with five different base modifications [^1^](#_ENREF_1)^,^[^2^](#_ENREF_2). Nucleotide bases were introduced in different numbers to increase the overall sequence diversity of the samples, thus improving the quality of the sequenced data. Bacterial DNA samples were divided into six groups and amplified using the six different primers. However, since ITS1 sequences are quite diverse across the fungal species [^3^](#_ENREF_3), this approach was not used for fungal ITS1 amplification.

Primer sequences for amplification of bacterial 16S rRNA V3 region are as below (the base inclusions are marked in bold):

The underlined regions in all the primer sequences are the Illumina Nextera XT adapter overhangs, whereas the non-underlined regions are the primer sequences known to target eubacterial 16S rRNA V3 region or fungal ITS1 region, respectively.

1. 341F-ADA

5’ TCGTCGGCAGCGTCAGATGTGTATAAGAGACAGCCTACGGGAGGCAGCAG 3’

534R-ADA

5’ GTCTCGTGGGCTCGGAGATGTGTATAAGAGACAGATTACCGCGGCTGCTGGC 3’

1. 341F_ADA_1B

5’ TCGTCGGCAGCGTCAGATGTGTATAAGAGACAG**T**CCTACGGGAGGCAGCAG 3’

534R_ADA_1B

5’ GTCTCGTGGGCTCGGAGATGTGTATAAGAGACAG**C**ATTACCGCGGCTGCTGGC 3’

1. 341F_ADA_2B

5’ TCGTCGGCAGCGTCAGATGTGTATAAGAGACAG**CT**CCTACGGGAGGCAGCAG 3’

534R_ADA_2B

5’ GTCTCGTGGGCTCGGAGATGTGTATAAGAGACAG**CT**ATTACCGCGGCTGCTGGC 3’

1. 341F_ADA_3B

5’ TCGTCGGCAGCGTCAGATGTGTATAAGAGACAG**CAT**CCTACGGGAGGCAGCAG 3’

534R_ADA_3B

5’ GTCTCGTGGGCTCGGAGATGTGTATAAGAGACAG**ACT**ATTACCGCGGCTGCTGGC 3’

1. 341F_ADA_4B

5’ TCGTCGGCAGCGTCAGATGTGTATAAGAGACAG**TCAT**CCTACGGGAGGCAGCAG 3’

534R_ADA_4B

5’ GTCTCGTGGGCTCGGAGATGTGTATAAGAGACAG**CTAT**ATTACCGCGGCTGCTGGC 3’

1. 341F_ADA_5B

5’ TCGTCGGCAGCGTCAGATGTGTATAAGAGACAG**CTACT**CCTACGGGAGGCAGCAG 3’

534R_ADA_5B

5’ GTCTCGTGGGCTCGGAGATGTGTATAAGAGACAG**CATCT**ATTACCGCGGCTGCTGGC 3’

The optimized PCR conditions were: initial denaturation at 94 °C for 5 minutes, followed by 35 cycles of denaturation at 94 °C for 30 seconds, annealing at 69 °C for 30 seconds, extension at 72 °C for 30 seconds and a final extension cycle at 72 °C for 5 minutes. Paq5000 DNA polymerase (Agilent technologies, USA) was used and 5% DMSO was added to the master mix to enhance the concentration of amplified product from the metagenomic template.

The amplification of fungal ITS1 region was performed using Illumina Nextera XT adapter-ligated ITS1 region-specific primers, ITS1-ADA-F and ITS1-ADA-R [^4^](#_ENREF_4)^,^[^5^](#_ENREF_5).

Primer sequences for amplification of fungal ITS1 region:

ITS1-ADA-F

5’ TCGTCGGCAGCGTCAGATGTGTATAAGAGACAGCTTGGTCATTTAGAGGAAGTAA 3’
ITS1-ADA-R

5’ GTCTCGTGGGCTCGGAGATGTGTATAAGAGACAGGCTGCGTTCTTCATCGATGC 3’

The optimized PCR conditions were: initial denaturation at 95 °C for 15 minutes (polymerase was added after this stage), followed by 35 cycles of denaturation at 94 °C for 1 minute, annealing at 66.5 °C for 2 minutes, extension at 72 °C for 2 minutes and a final extension cycle at 72 °C for 10 minutes. Paq5000 DNA polymerase (Agilent technologies, USA) was used and a final concentration of 2.5 mM MgCl_2_ was added to the PCR master mix to enhance the amplification of ITS1 region.

**Gene quantification**

Gene quantification was carried out as described previously [^6^](#_ENREF_6). In brief, the high-quality reads were aligned against the combined gene catalogue using SOAP2 in SOAP aligner [^7^](#_ENREF_7) for fungi and Bowtie2 for bacteria [^8^](#_ENREF_8). For sequence-based profiling, two types of alignments were considered: (i) both pairs of a paired-end read completely map to a gene, or (ii) one end of the paired-end read maps to a gene and the other end remains unmapped. For both cases, the mapped read was counted as one copy. Further, the read count was normalized based on the length of the gene and was calculated as: $bi=\frac{xi}{Li}$

The relative abundance of a gene within the sample was calculated as:$ai=\frac{bi}{\sum j bj}=\frac{\frac{xi}{Li}}{\sum j\frac{xj}{Lj}}$

*a_i_*: relative abundance of gene 𝑖 in sample S; *x_i_*: the number of times a gene *i* can be detected in sample S (the number of mapped reads); *L_i_*: length of gene i; *b_i_*: copy number of gene *i* in sequenced data from sample S.

**Fasta sequences of OTUs corresponding to ‘species close to M. restricta’**

>seq_0

CATTAGTGAAGATTTGGGCAGGCCATACGGACGCCAAAAAGTGTCCCTGGCCGCCTACACCCACTATACATCCACAAACCCGTGTGCGCTGTCTTGGAGAAAGGCTTCAGAGAAGTTTTTTGTGGCCTCTCTTGGGGTCTTTCTTCGCTACAAACTCGAATGGTTAGTATGAACGTGGAACTTGGTTGGACCGTCACTGGCCAACAAACTATACACAACTTTCGACAACGGATCTCTTGGTTCTCCCATCGATGAAGAACGCAGCGAAACGCGATAGGTAATGTGAATTGCAGAATTCCGTGAATCATCGAATCTTTGAACGCACCTTGCGCTCTATGGCAATCCGTAGAGCATGCCTGTTTGAGTGCCATGAAATCTCCCACCCCAAGCGGTTTTTAAATGAAACGGCTTGGCGGATGGGGTCTGGATGGGTGCCTCTGCCTGCGCTACCCTGCACAGGCTCGCCCGAAATGCATGAGCGCCTTGAGACACTTTGCATCCGCCTCTCTGTTTGGGAGGAGGCGGCCAAGCAGTGTTTTTCTCCTGGCATGGCATGATACGTCATTTGCTATGTCGCCTA

>seq_40

AGAGCATGTGAACTGAGACCATGGGCGGCCATACGGACGCCAAAAAGTGTCCCTGGCCGCCTACACCCACTATACATCCACAAACCCGTGTGCACTGTCTTGGAGAAAGGCTTCAGAGAAGTTTTTTGTGGCCTCTCTTGGGGTCTTTCTTCGCTACAAACTCGAATGGTTAGTATGAACGTGGAACTTGGTTGGACCGTCACTGGCCAACAAACTATACACAACTTTCGACAACGGATCTCTTGGTTCTCCCATCGATGAAGAACGCAGCGAAACGCGATAGGTAATGTGAATTGCAGAATTCCGTGAATCATCGAATCTTTGAACGCACCTTGCGCTCTATGGCAATCCGTAGAGCATGCCTGTTTGAGTGCCATGAAATCTCCCACCCCAAGCGGTTTTTACATGAAACGGCTTGGCGGATGGGGTCTGGATGGGTGCCTCTGCCTGCGCTACCTAGCACAGGCTCGCCCGAAATGCATGAGCGCCTTGAGACACTTTGCATCCGCCTCTCTGTTTGGGAGGAGGCGGCCAAGCAGTGTTTTTCTCCTGGCATGGCATGATACGTCATTTGCTATGTCGCCTAAAGGAGGAATGTTTGGTTGTGTCTGCGTGTGCTTCGAACTTGCCTCTGTGGCACATCCCAATTTCACTTCTGGTCTCAAATCAGGTAGGATCACCCGCTGAACTTAAGCATATCAATAAGCGGAGGAAAAGAAACTAACAGGATTCCCCTAGTAACGGCGAGCGAAGCTGGAAGAGCTCAAATTTGAAAGCTGGCGTCTTCGGCGTCCGCGTTGTAATCTCGAGACGTGTTTTCCGTGCGGCTCTATGGACAAGTCCCTTGGAACAGGGTATCGTAGAGGGTGAAAATCCCGTACTTGCCATGGAAATACCGTGCTTTGTGATACACGCTCCAAGAGTCGAGTAGATTGAGATAGCTGCTCCAAGTGGGTGGTAAACTCCATCTAAGCTAATAACGGGGAGAGACCCATAGCGAACAAGTACGTGGAGGAAAGAGACAACCCCTTCGTAAAGAGAGTTAAAATACGTAAATGTGCCAAAGGGAACCATTGGAGTCTGCATTCTGCTGAGAACTCAACCTGGCTTTTGCTTGGGGTATTTTCCGGACACAG

>seq_41

GGATCATTAGTGAAGATTTGGGCAGGCCATACGGACGCCAAAAAAGTGTCCCTGGCCGCCTACACCCACTATACATCCACAAACCCGTGTGCACTGTCTTGGAGAAAGGCTTCTTGAGAAGTTATGTGACCTCTCTTGGAGGTCTTTCTTCGCTACAAACTCGAATGGTTAGTATGAACGTGGAACTTGGTTGGACCGTCACTGGCCAACAAACTATACACAACTTTCGACAACGGATCTCTTGGTTCTCCCATCGATGAAGAACGCAGCGAAACGCGATAGGTAATGTGAATTGCAGAATTCCGTGAATCATCGAATCTTTGAACGCACCTTGCGCTCGATGGCAATCCGTAGAGCATGCCTGTTTGAGTGCCATGAAATCTCCCACCCCAAGCGGTTTTTGAATGAAACGGCTTGGCGGATGGGGTCTGGATGGGTGCCTCTGCCTGTGCTAAACCTTAGCCCACAGGCTCGCCCGAAATGCATGAGCGCCTTGGGACACTTTGCATCCGCCTCTCTTGTGGGAGGAGGCGGCCAAGCAGTGTTTTTCTCCAGGCATGGCATGATACGTCATTTGCTATGTCGTCCAATGGAGGAATGTTTG

>seq_59

ATCATTAGTGAAGATTTGGGCAGGCCATACGGACGCCACAAAGTGTCCCTGGCCGCCTACACCCACTATACATCCACAAACCCGTGTGCACTGTCCTTGGAAAGGGCATTTGGAGAGAAAAAGAAAGAGAAGCGAGAGCAGTGCGATCCGTCGCGTTGAGCGTTTTCTCTGACTTGGTCTCTCTGAGGCCCTTTCCTTGCTACAAACTCGAATGGTTAGTATGAACGTGGAACTTGGTTGGACCGTCACTGGCCAACAAACTATACACAACTTTCGACAACGGATCTCTTGGTTCTCCCATCGATGAAGAACGCAGCGAAACGCGATAGGTAATGTGAATTGCAGAATTCCGTGAATCATCGAATCTTTGAACGCACCTTGCGCTCTATGGCAATCCGTAGAGCATGCCTGTTTGAGTGCCGTGAAATCTCCCACCCCAAGCGGTTTTTACATGAAACGGCTTGGCGGATGGGGTCTGGATGGGTGCCTCTGCCTGCGCTACCTAGCACAGGCTCGCCCGAAATGCATGAGCGCCTTGAGACACTTTGCATCCGCCTCTCTGTTTGGGAGGAGGCGGCCAAGCAGTGTTTTTCTCCTGGCATGGCATGATACGTCATTTGCTATGTCGCCTAAAGGAGGAATGTTTGGTTGTGTCTGCGTGTGCTTCGAACTTGCCTCTGTGGCACATCCCAATTTCACTTCTGGTCTCAAATCAGGTAGGATCACCCGCTGAACTTAA

>seq_79

AAGTCGTAACAAAGGTTTCTGTAGGTGAACCTGCAGAAGGATCATTAGTGAAGATTTGGGCTGGCCATACGGACGCCAAAAAGTGTCCCTGGCCGCCCTACCACCCCACTATACATCCACAAACCCGTGTGCACTGTCTTGGAGAAAGGCTTCAGAGAAGTTTTTTGTGGCCTCTCTTGGGGTCTTTCTTCGCTCCAAACTCGAATGGTTAGTATGAACGTGGAACTTGGTTGGACCGTCACTGGCCAACAAACTATACACAACTTTCGACAACGGATCTCTTGGTTCTCCCATCGATGAAGAACGCAGCGAAACGCGATAGGTAATGTGAATTACAGAATTCCGTGAATCATCGAATCTTTGAACGCACCTTGCGCTCTATGGCAATCCGTAGAGCATGCCTGTTTGAGTGCCATGAAATCTCCCACCCCAAGCGGTTTTTACATGAAACGGCTTGGCGGATGGGGTCTGGATGGGTGCCTCTGCCTGCGCTACCTAGCACAGGCTCGCCCGAAATGCATGAGCGCCTTGAGACACTTTGCATCCGCCTCTCTGTTTGGGAGGAGGCGGCCAAGCAGTGTTTTTCTCCTGGCATGGCATGATACGTCATTTGCTATGTCGCCTAAAGGAGGAATGTTTGGTTGTGTCTGCGTGTGCTTCGAACTTGCCTCTGTGGCACATCCCAATTTCACTTCTGGTCTCAAATCAGGTAGGATCACCCGCTGAACTTAA

>seq_86

AAAGTCGTAACAAGGTTTCTGTAGGTGAACCTGCAGAAGGATCATTAGTGAAGATTTGGGCAGGCCATGCGGACGCCACAAAGTGTCCCTGGCCGCCTACACCCACTATACATCCACAAACCCGTGTGCACTGTCCTTGGAAAGGGCATTTGGAGAGAAAAAGGAAGAGAGAAGCGAGAACAGTGCGATCCGTCGCGTTGGGCGTTGATCTCTGGCTTGGTCTCTCTGAGGCCCTTCCCTCGCTACAAACTCGAATGGTTAGTATGAACGTGGAACTTGGTTGGACCGTCACTGGCCAACAAACTATACACAACTTTCGACAACGGATCTCTTGGTTCTCCCATCGATGAAGAACGCAGCGAAACGCGATAGGTAATGTGAATTGCAGAATTCCGTGAATCATCGAATCTTTGAACGCACCTTGCGCTCTATGGCAATCCGTAGAGCATGCCTGTTTGAGTGCCATGAAATCTCCCACCCCAAGCGGTTTTTAAATGAAACGGCTTGGCGGATGGGGTCTGGATGGGTGCCTCTGCCTGCGCTACCTAGCACAGGCTCGCCCGAAATGCATGAGCGCCTTGAGACACTTTGCATCCGCCTCTCTGTTTGGGAGGAGGCGGCCAAACAGTGTTTTTCTCCTGGCATGGCATGATACGTCATTTGCTATGTCACCTAAAGGAGGAATGTTTGGTTGTGTCTGCCTGTGCTTCAAACCTG

Taxonomic lineage

seq_0 k__Fungi; p__Basidiomycota; c__Malasseziomycetes; o__Malasseziales; f__Malasseziaceae; g__Malassezia; s__close_to_Malassezia restricta

seq_40 k__Fungi; p__Basidiomycota; c__Malasseziomycetes; o__Malasseziales; f__Malasseziaceae; g__Malassezia; s__close_to_Malassezia restricta

seq_41 k__Fungi; p__Basidiomycota; c__Malasseziomycetes; o__Malasseziales; f__Malasseziaceae; g__Malassezia; s__close_to_Malassezia restricta

seq_59 k__Fungi; p__Basidiomycota; c__Malasseziomycetes; o__Malasseziales; f__Malasseziaceae; g__Malassezia; s__close_to_Malassezia restricta

seq_79 k__Fungi; p__Basidiomycota; c__Malasseziomycetes; o__Malasseziales; f__Malasseziaceae; g__Malassezia; s__close_to_Malassezia restricta

seq_86 k__Fungi; p__Basidiomycota; c__Malasseziomycetes; o__Malasseziales; f__Malasseziaceae; g__Malassezia; s__close_to_Malassezia restricta

***Supplementary References***

1 Wang, Y. & Qian, P. Y. Conservative fragments in bacterial 16S rRNA genes and primer design for 16S ribosomal DNA amplicons in metagenomic studies. *PLoS One* **4**, e7401, doi:10.1371/journal.pone.0007401 (2009).

2 Soergel, D. A., Dey, N., Knight, R. & Brenner, S. E. Selection of primers for optimal taxonomic classification of environmental 16S rRNA gene sequences. *The ISME journal* **6**, 1440-1444, doi:10.1038/ismej.2011.208 (2012).

3 Wang, X. C. *et al.* ITS1: a DNA barcode better than ITS2 in eukaryotes? *Molecular ecology resources* **15**, 573-586 (2015).

4 Tonge, D. P., Pashley, C. H. & Gant, T. W. Amplicon–Based Metagenomic Analysis of Mixed Fungal Samples Using Proton Release Amplicon Sequencing. *PLoS One* **9**, e93849 (2014).

5 Ihrmark, K. *et al.* New primers to amplify the fungal ITS2 region–evaluation by 454-sequencing of artificial and natural communities. *FEMS microbiology ecology* **82**, 666-677 (2012).

6 Qin, J. *et al.* A metagenome-wide association study of gut microbiota in type 2 diabetes. *Nature* **490**, 55-60, doi:10.1038/nature11450 (2012).

7 Li, R. *et al.* SOAP2: an improved ultrafast tool for short read alignment. *Bioinformatics* **25**, 1966-1967, doi:10.1093/bioinformatics/btp336 (2009).

8 Langmead, B. & Salzberg, S. L. Fast gapped-read alignment with Bowtie 2. *Nat Methods* **9**, 357-359, doi:10.1038/nmeth.1923 (2012).
