# Supplementary material for: Longitudinal study of the scalp microbiome suggests coconut oil to enrich healthy scalp commensals
Source: Sci Rep. 2021 Mar 31;11:7220. doi: 10.1038/s41598-021-86454-1 (PMC8012655; doi:10.1038/s41598-021-86454-1)
Supplement: Supplementary file 2 — Supplementary Information 2. [file 41598_2021_86454_MOESM2_ESM.docx]

**Supplementary Figures**

**Fig. S1.** (a) Shannon diversity index for fungal population observed in the three phases. (b to d) Taxonomic composition of fungal species observed in healthy and dandruff scalp at the baseline (*t*=1). Fungal species with ≥1% abundance (in average) are plotted in bar plots representing the abundant species in each sample. Top five species in each group are plotted in the pie plots. (e) Differences in the ratio of *Malassezia restricta* to *Malassezia globosa* in the healthy and dandruff groups after treatment phase (*, *p*≤0.05).

**Fig. S2. Abundance of fungal species at the treatment phase (*t*=2).** Fungal species with ≥1% abundance (in average) are plotted in bar plots representing the abundant species in each sample. Top five species in each group are plotted in the pie plots.

**Fig. S3. Abundance of fungal species at the relapse phase (*t*=3).** Fungal species with ≥1% abundance (in average) are plotted in bar plots representing the abundant species in each sample. Top five species in each group are plotted in the pie plots.

**Fig. S4.** (a) Shannon diversity index for bacterial population observed in the three phases. (b) Unweighted UniFrac distances observed in the bacterial population at the three phases (***, p≤0.001). (c to e) Taxonomic composition of bacterial species observed in healthy and dandruff scalp at the baseline (*t*=1). Fungal species with ≥1% abundance (in average) are plotted in bar plots representing the abundant species in each sample. Top five species in each group are plotted in the pie plots.

**Fig. S5.** (a) Differences in the ratio of *Cutibacterium acnes* to *Staphylococcus epidermidis* in the healthy and dandruff groups after treatment phase (*, *p*≤0.05). Abundance of bacterial species at the treatment phase (*t*=2). (b to e) Fungal species with ≥1% abundance (in average) are plotted in bar plots representing the abundant species in each sample. Top five species in each group are plotted in the pie plots.

**Fig. S6. Abundance of bacterial species at the relapse phase (*t*=3).** Fungal species with ≥1% abundance (in average) are plotted in bar plots representing the abundant species in each sample. Top five species in each group are plotted in the pie plots.

**Fig. S7.** (a) Differentially abundant (*p*≤0.05) fungal pathways between healthy and dandruff scalp at baseline. (b) Differentially abundant bacterial KEGG pathways related to amino acids, vitamins and cofactors in healthy and dandruff scalp at the baseline (p≤0.05). (c) Significant variations in the KOs related to biotin metabolism observed between different groups (*p*≤0.05, Wilcoxon test). (d) Significant variations in the KOs related to biotin transport observed between different groups (*p*≤0.05, Wilcoxon test).

**Table S1. Results for repeated measures ANOVA (xlsx).**

**Table S2. Subject grouping and sequencing statistics (xlsx).**
