# Supplementary figures and images for: Longitudinal study of the scalp microbiome suggests coconut oil to enrich healthy scalp commensals
Source: Sci Rep. 2021 Mar 31;11:7220. doi: 10.1038/s41598-021-86454-1 (PMC8012655; doi:10.1038/s41598-021-86454-1)

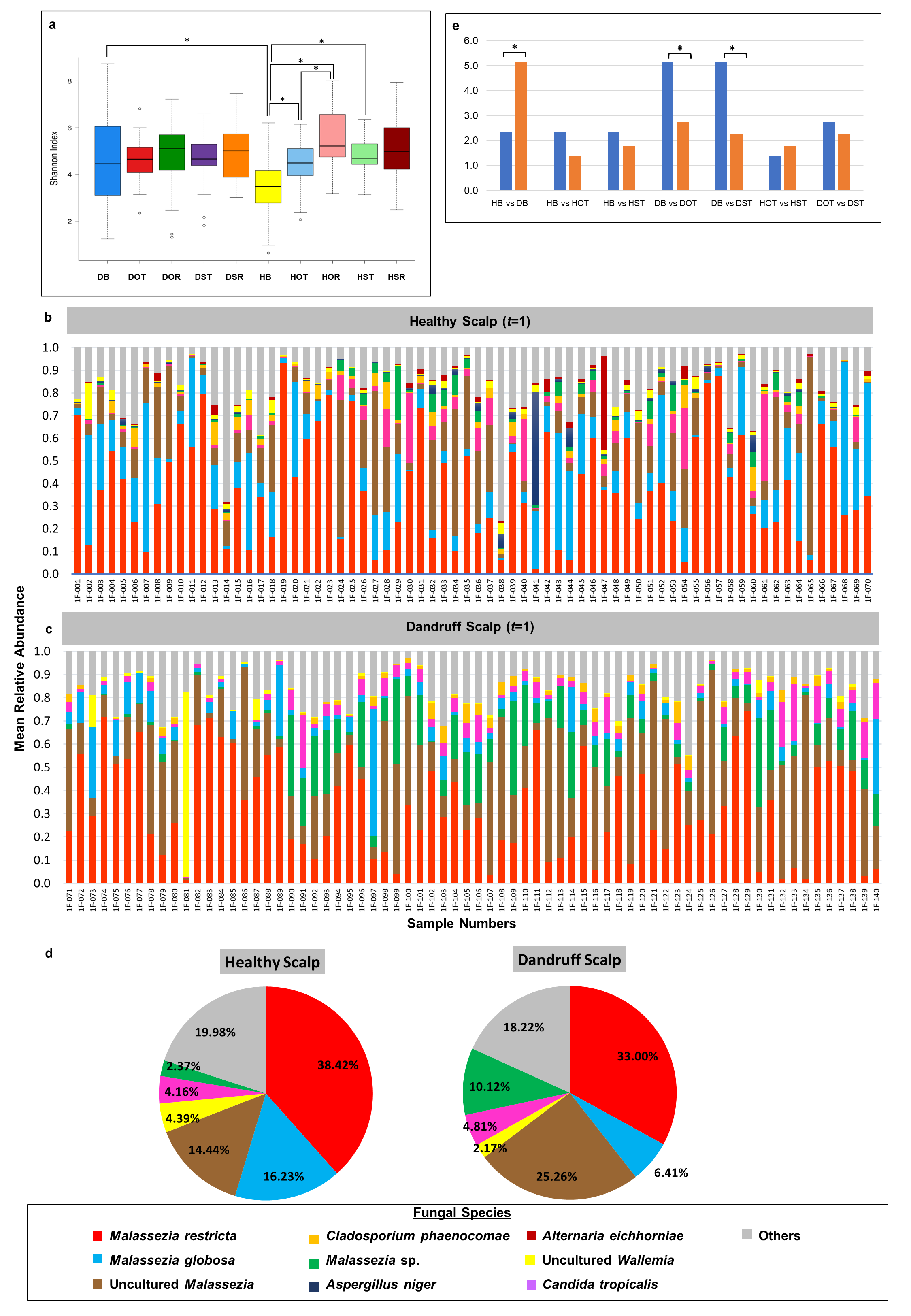

Supplement: Supplementary file 5 — Supplementary Information 5. [file 41598_2021_86454_MOESM5_ESM.tif]

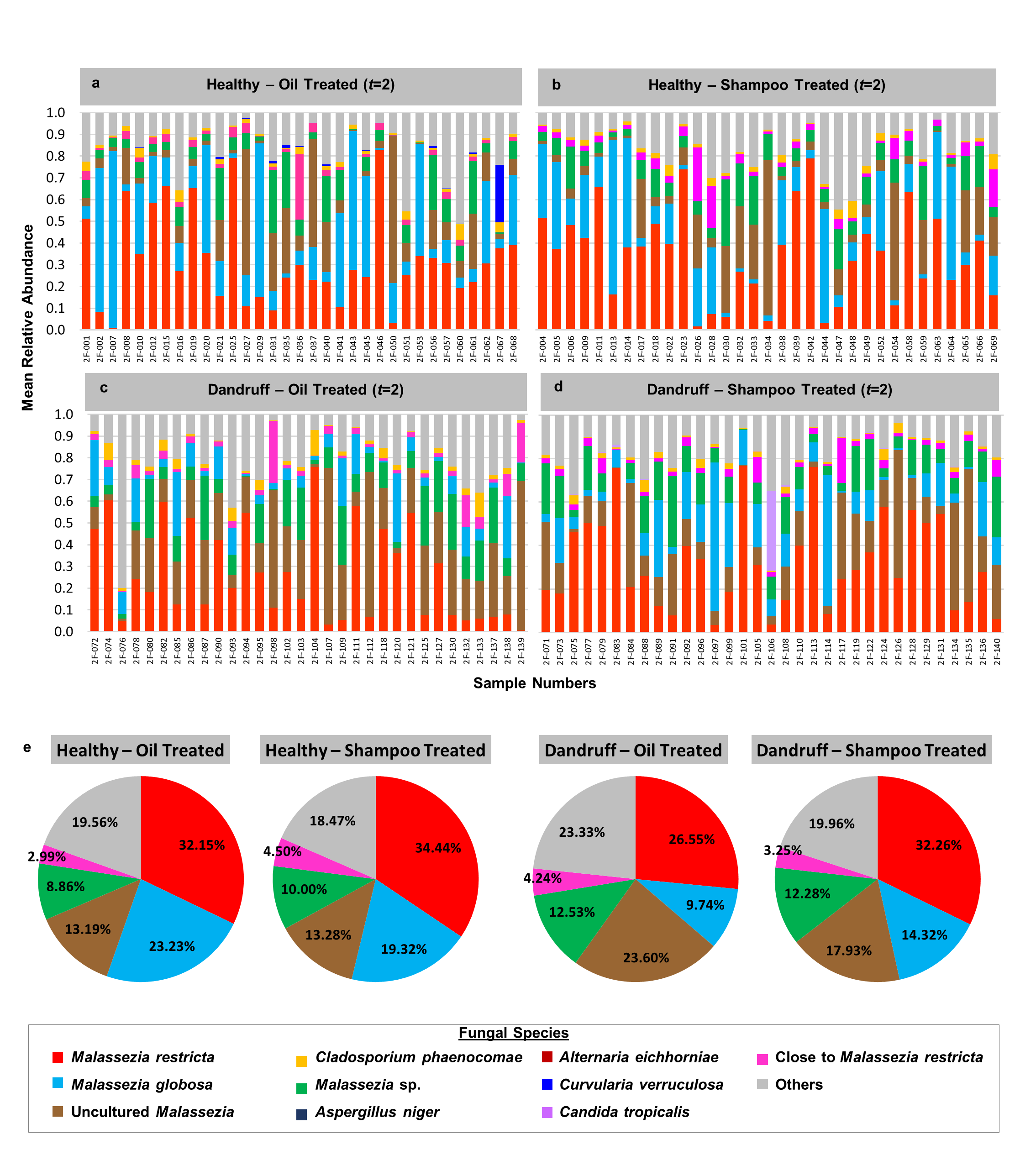

Supplement: Supplementary file 6 — Supplementary Information 6. [file 41598_2021_86454_MOESM6_ESM.tif]

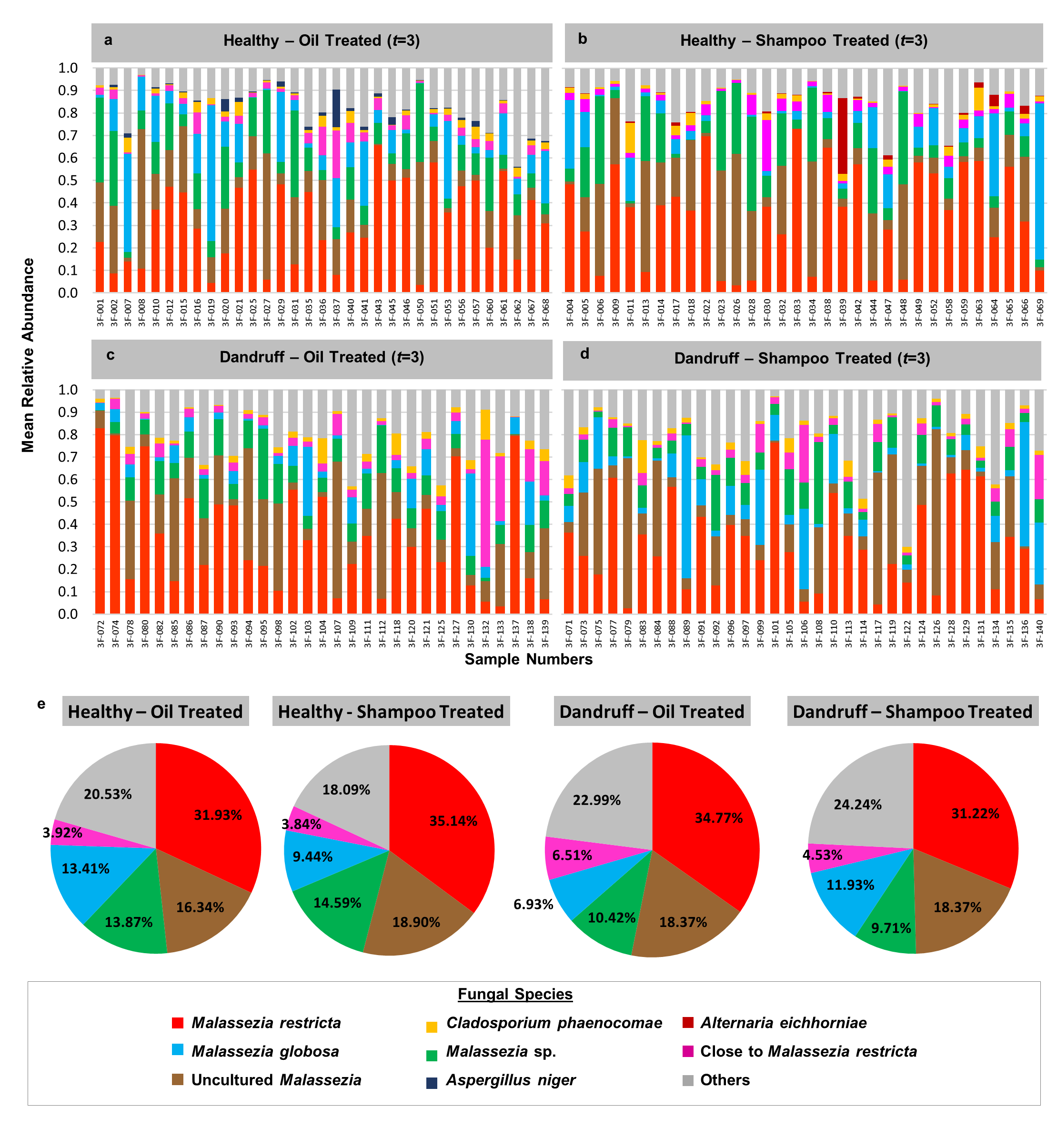

Supplement: Supplementary file 7 — Supplementary Information 7. [file 41598_2021_86454_MOESM7_ESM.tif]

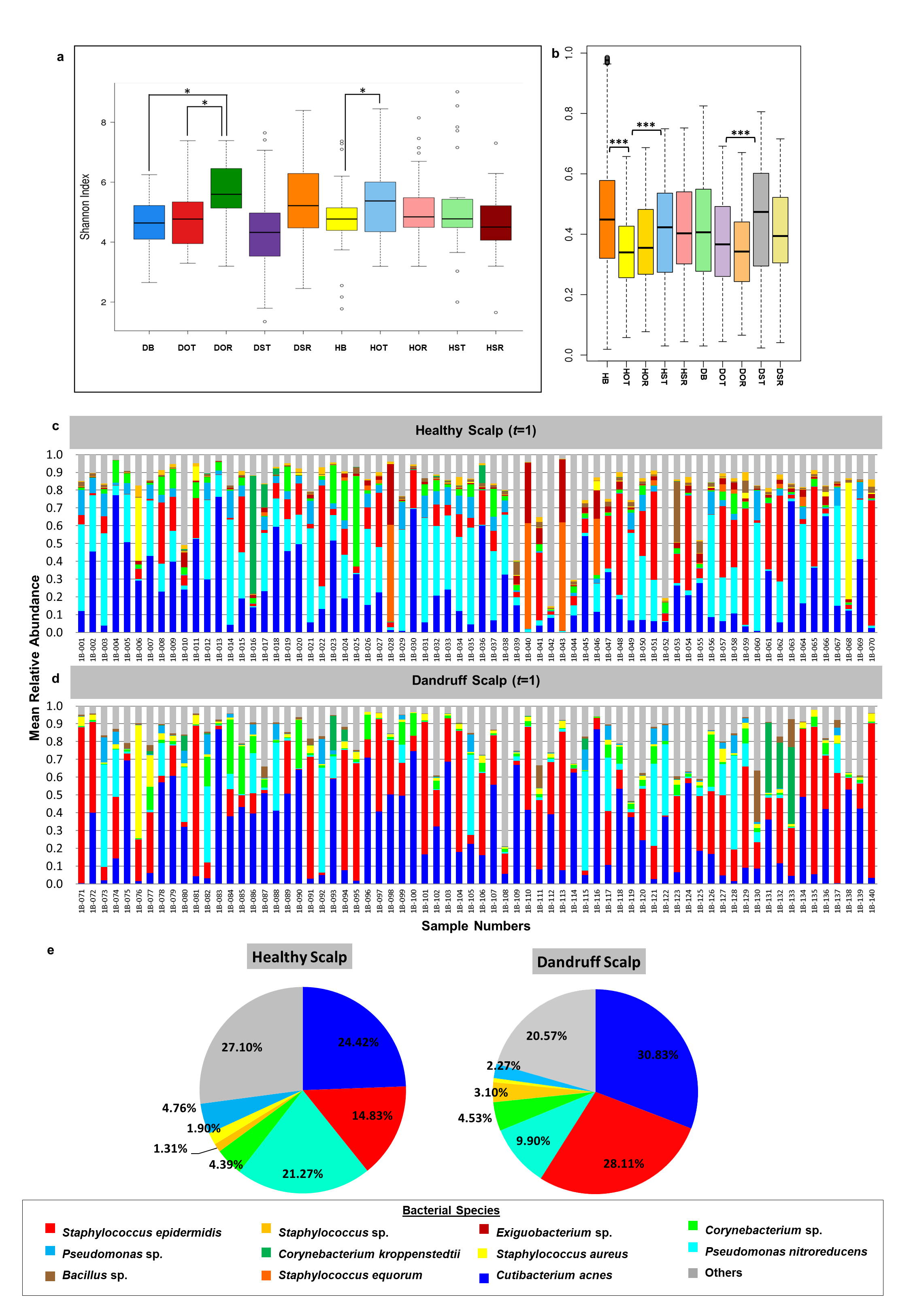

Supplement: Supplementary file 8 — Supplementary Information 8. [file 41598_2021_86454_MOESM8_ESM.tif]

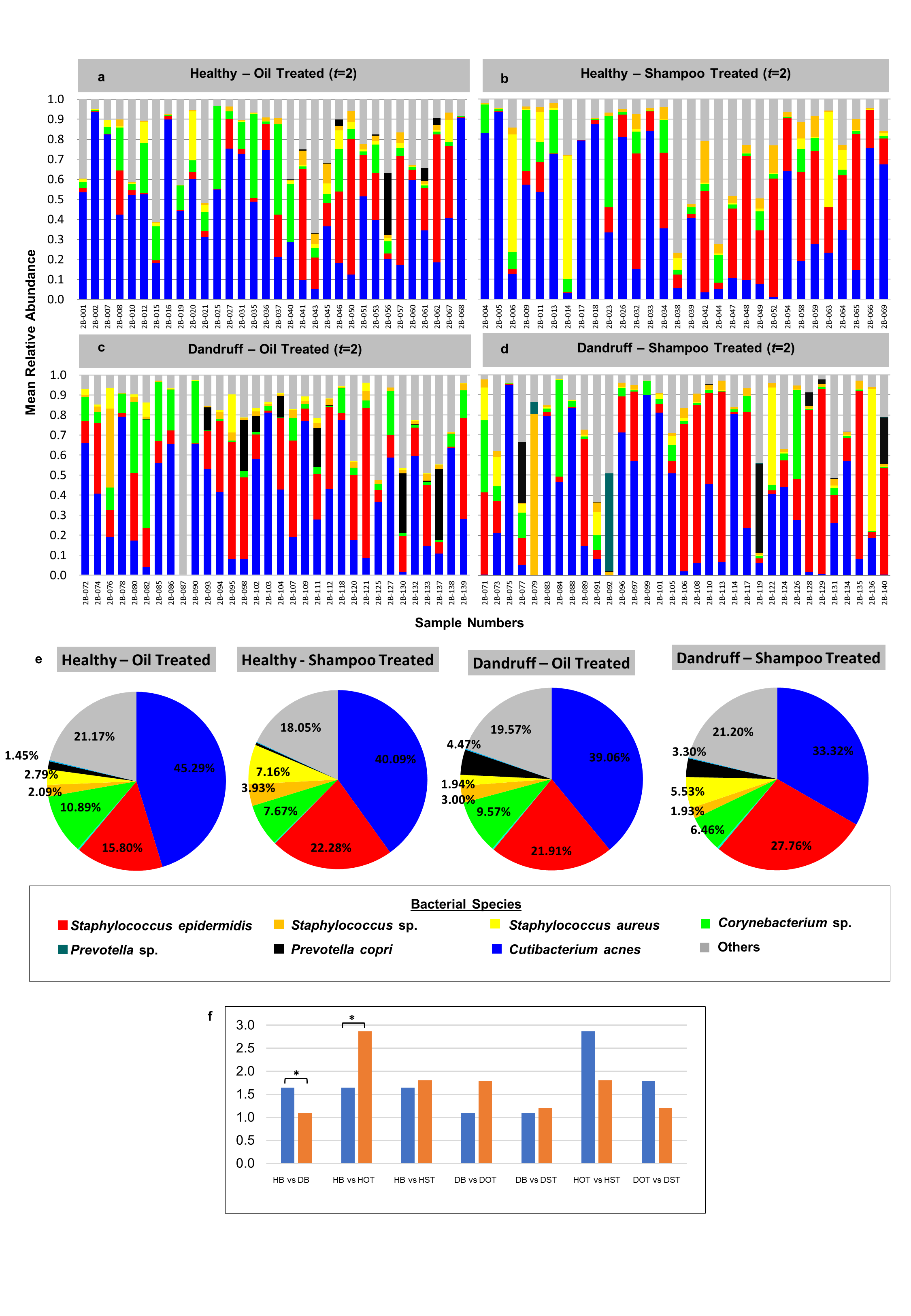

Supplement: Supplementary file 9 — Supplementary Information 9. [file 41598_2021_86454_MOESM9_ESM.tif]

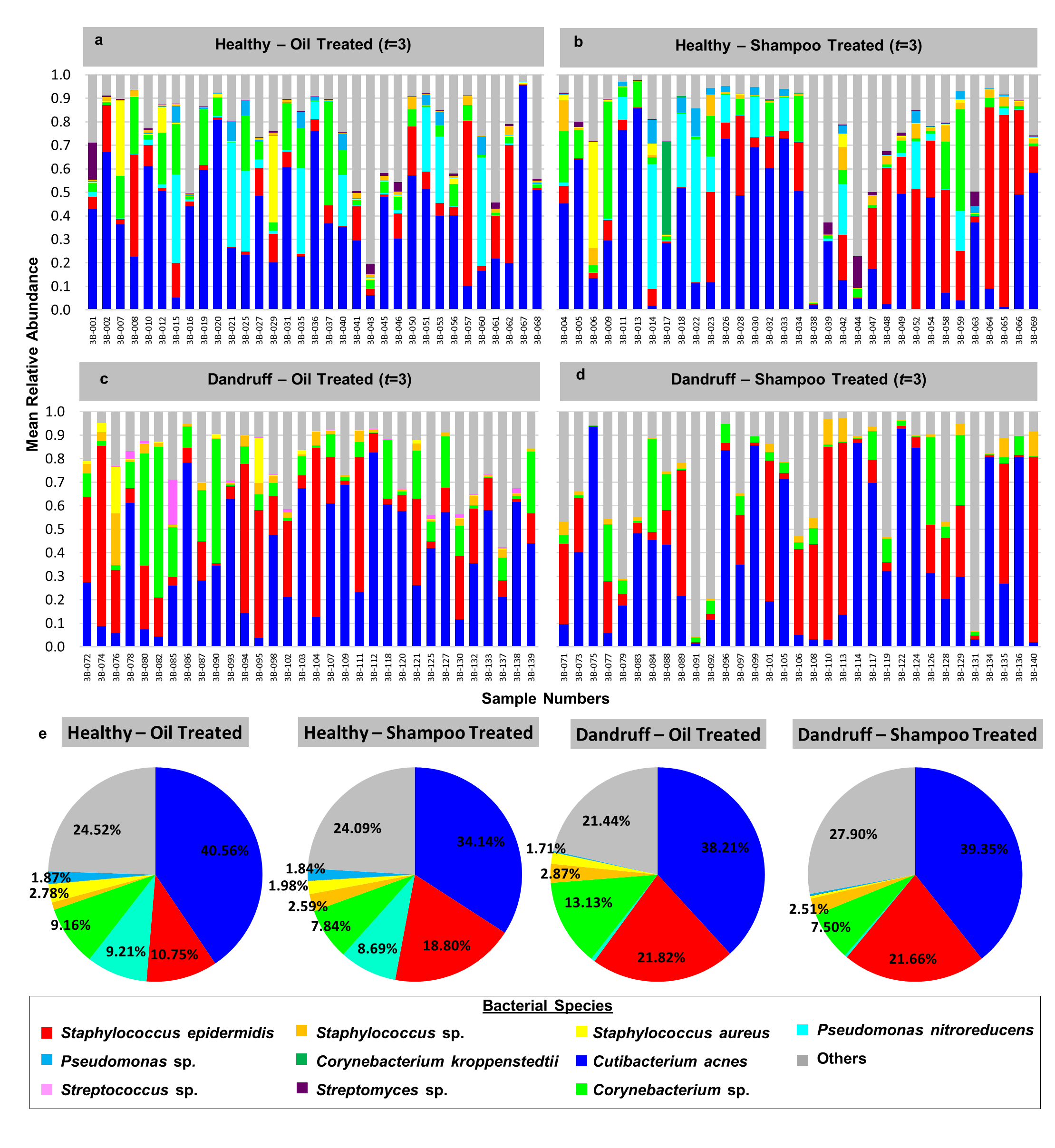

Supplement: Supplementary file 10 — Supplementary Information 10. [file 41598_2021_86454_MOESM10_ESM.tif]

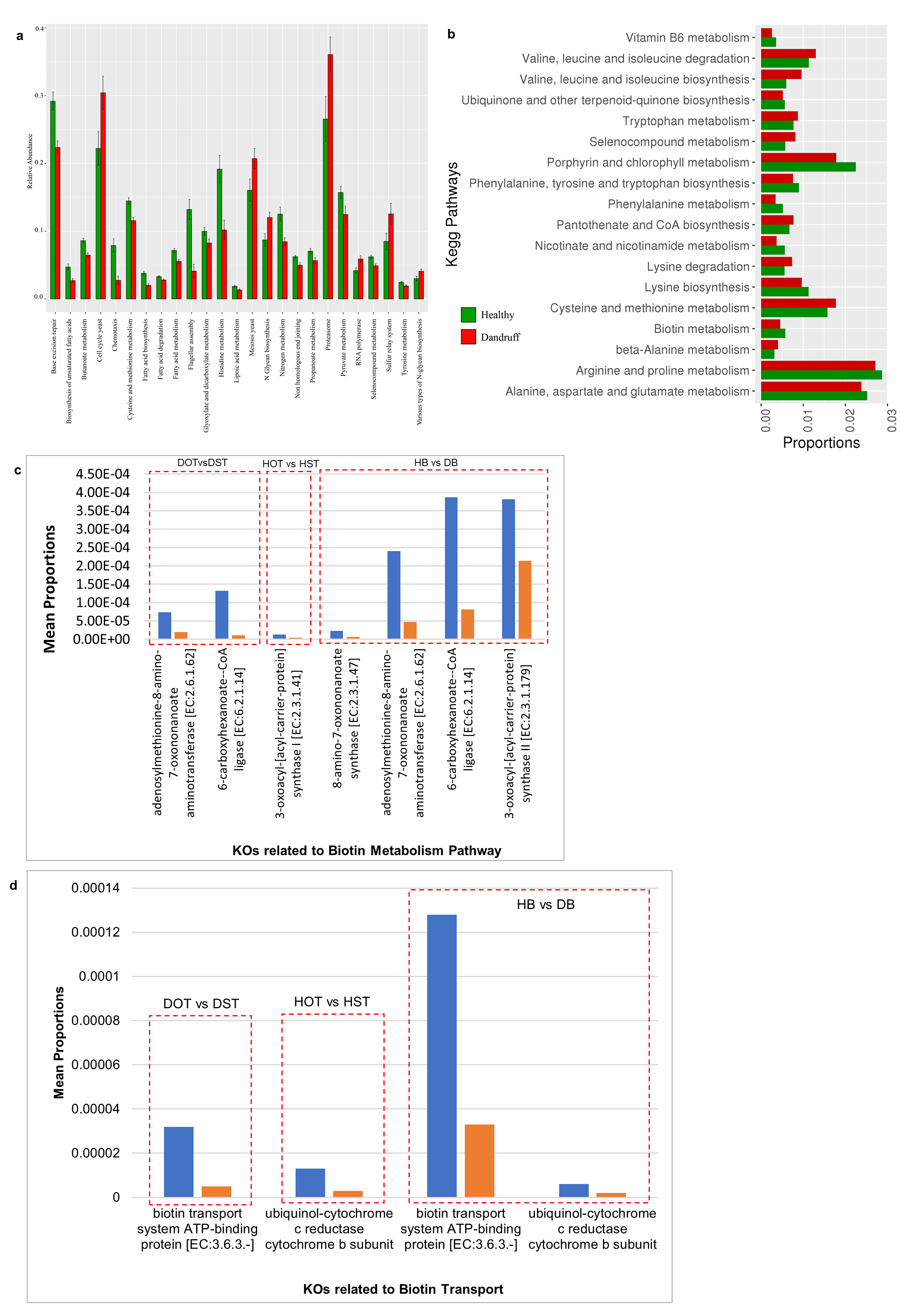

Supplement: Supplementary file 11 — Supplementary Information 11. [file 41598_2021_86454_MOESM11_ESM.tif]
